# Synthesis, Molecular Docking, and Dynamic Simulation Targeting Main Protease (Mpro) of New, Thiazole Clubbed Pyridine Scaffolds as Potential COVID-19 Inhibitors

**DOI:** 10.3390/cimb45020093

**Published:** 2023-02-07

**Authors:** Adel Alghamdi, Amr S. Abouzied, Abdulwahab Alamri, Sirajudheen Anwar, Mukhtar Ansari, Ibrahim Khadra, Yasser H. Zaki, Sobhi M. Gomha

**Affiliations:** 1 Pharmaceutical Chemistry Department, Faculty of Clinical Pharmacy, Al Baha University, Al Baha P.O. Box 1988, Saudi Arabia; 2 Department of Pharmaceutical Chemistry, College of Pharmacy, University of Hail, Hail 81442, Saudi Arabia; 3 Department of Pharmaceutical Chemistry, National Organization for Drug Control and Research (NODCAR), Giza 12311, Egypt; 4 Department of Pharmacology and Toxicology, College of Pharmacy, University of Hail, Hail 81442, Saudi Arabia; 5 Department of Clinical Pharmacy, College of Pharmacy, University of Hail, Hail 81442, Saudi Arabia; 6 Strathclyde Institute of Pharmacy and Biomedical Sciences, University of Strathclyde, 161 Cathedral Street, Glasgow G4 0RE, UK; 7 Department of Chemistry, Faculty of Science, Beni-Suef University, Beni-Suef 62514, Egypt; 8 Department of Chemistry, Faculty of Science, Islamic University of Madinah, Madinah 42351, Saudi Arabia; 9 Department of Chemistry, Faculty of Science, Cairo University, Giza 12613, Egypt

**Keywords:** hydrazonoyl chlorides, acetyl pyridines, thiazoles, molecular docking, schiff bases, COVID-19

## Abstract

Many biological activities of pyridine and thiazole derivatives have been reported, including antiviral activity and, more recently, as COVID-19 inhibitors. Thus, in this paper, we designed, synthesized, and characterized a novel series of *N*-aminothiazole-hydrazineethyl-pyridines, beginning with a *N*′-(1-(pyridine-3-yl)ethylidene)hydrazinecarbothiohydrazide derivative and various hydrazonoyl chlorides and phenacyl bromides. Their Schiff bases were prepared from the condensation of N-aminothiazole derivatives with 4-methoxybenzaldehyde. FTIR, MS, NMR, and elemental studies were used to identify new products. The binding energy for non-bonding interactions between the ligand (studied compounds) and receptor was determined using molecular docking against the SARS-CoV-2 main protease (PDB code: 6LU7). Finally, the best docked pose with highest binding energy (**8a** = −8.6 kcal/mol) was selected for further molecular dynamics (MD) simulation studies to verify the outcomes and comprehend the thermodynamic properties of the binding. Through additional in vitro and in vivo research on the newly synthesized chemicals, it is envisaged that the achieved results will represent a significant advancement in the fight against COVID-19.

## 1. Introduction

Recently, antiviral, chemotherapeutic drugs are ineffective in clinic settings. This is a result of the development of a number of significant viral infections, which has resulted in widespread human disease and mortality. Coronaviruses (CoV) are a large group of viruses that affect a wide variety of animals. They have caused serious and deadly respiratory infections in both humans and animals, such as severe acute respiratory syndrome coronavirus 2 (SARS-CoV-2) and the Middle East respiratory syndrome coronavirus (MERS-CoV) [1,2,3,4,5]. The World Health Organization (WHO) reported the development of coronavirus disease 2019 (COVID-19). In terms of the persons afflicted and the geographic scope of the outbreak, COVID-19 has significantly exceeded SARS and MERS [6]. Therefore, new antiviral candidates that are based on diverse heterocyclic compounds are urgently desired and are unquestionably necessary for the treatment of numerous deadly viral infections [7,8,9,10].

**Figure 1:**
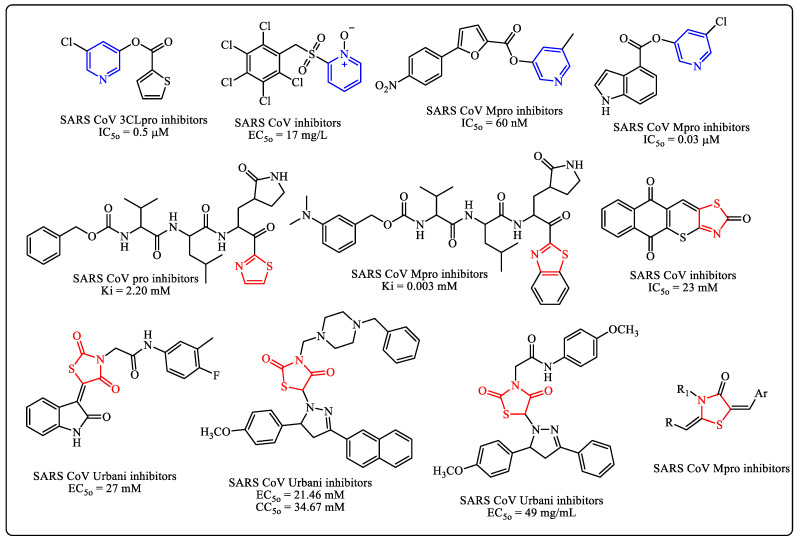
Pyridine and thiazole derivatives as potential coronaviruses.

Pyridine compounds are obtaining importance in the field of medicinal chemistry because of the broad spectrum of their physiological activities, including antiviral activity [11,12,13,14,15], especially against COVID-19 (Figure 1) [16,17,18,19,20]. On the other hand, thiazoles are present in many drugs or prodrugs. Thiazoles have been researched for their potential to combat coronaviruses over the past ten years (Figure 1) [21,22,23,24,25].

Due to the aforementioned reasons, and as a part of our ongoing research to develop new, bioactive heterocycles [8,10,26,27,28,29,30,31,32,33,34,35,36], we report herein the simple and efficient synthesis of new series of pyridine–thiazole hybrids utilizing molecular docking and molecular dynamics simulation (MDS), which demonstrate the ability of the studied compounds to successfully bind to the SARS-CoV-2 main protease, to explore their antiviral properties against the SARS-CoV-2 main protease (Mpro).

## 2. Experimental

### 2.1. Chemistry

Synthesis of thiazole derivatives **6a**–**e** and **13a**–**c**.

Catalytic amounts of TEA were added into a solution of N’-(1-(pyridin-3-yl) ethylidene)hydrazine-carbothiohydrazide (**3**) (0.209 g, 1 mmol) and the appropriate hydrazonoyl chlorides **4a**–**e** or α-bromoketones **11a**–**c** (1 mmol for each) in DMF (20 mL). The reaction mixture was refluxed for 3–6 h. Finally, the formed precipitate was isolated and recrystallized from the suitable solvent to yield the compounds **6a**–**e** or **13a**–**c,** respectively.

4-Methyl-5-phenyldiazenyl-2-((1-(pyridin-3-yl)ethylidene)hydrazineylidene)thiazol-3(2H)-amine **(6a).** Red solid, 78% yield, m.p. 155–157 °C (EtOH); IR (KBr): *v* 3426, 3271 (NH_2_), 1606 (C=N) cm^−1^; ^1^H-NMR (DMSO-*d*_6_): *δ* = 2.38 (s, 3H, CH_3_), 2.63 (s, 3H, CH_3_), 5.81 (s, 2H, NH2), 7.18–7.66 (m, 6H, Ar-H and Pyr-H5), 8.20 (d, 1H, Pyr-H4), 8.58 (d, 1H, Pyr-H6), 9.02 (s, 1H, Pyr-H2) ppm; ^13^C-NMR (DMSO-*d*_6_): *δ* = 12.49, 14.14 (CH_3_), 101.16, 119.08, 123.68,129.26, 129.32, 129.51, 133.69, 133.85, 134.42, 137.20, 148.41, 150.19, 155.82 (Ar-C and C=N)ppm; MS *m/z* (%): 351 (M^+^, 58). Anal. Calcd for C_17_H_17_N_7_S (351.13): C, 58.10; H, 4.88; N, 27.90. Found: C, 58.03; H, 4.66; N, 27.79%.

Synthesis of Schiff bases **8a,d** and **14a**–**c**.

A catalytic amount of HCl concentration was added to a solution of 4-methoxybenzaldehyde (**7**) (1.36 g, 10 mmol), and the appropriate **8a,d** or **13a**–**c** (1 mmol for each) was added to DMF (20 mL). The reaction mixture was refluxed for 2–4 h. Finally, the formed precipitate was recrystallized from the suitable solvent to yield compounds **6a**–**e** or **13a**–**c,** respectively.

Physical and spectral data of all synthesized compounds **6a**–**e**, **13a**–**c, 8a,d** and **14a**–**c** are frond in the supporting information file.

### 2.2. Docking Method

The newly synthesized compounds were subjected to docking tests using the molecular operating environment 2019.012 suite (Montreal, QC, Canada) [37] to ascertain how well they bound and to propose their mechanism of action as SARS-CoV-2 Mpro inhibitors in comparison to the co-crystallized inhibitor (N3), which was used as a reference standard. Energy was minimized and a partial charge was added to the freshly synthesized molecules inside the MOE window [38,39]. The synthesized compounds were then combined with (N3) in one database and stored as an MDB file that could be transferred into the ligand icon during the binding stage. The Protein Data Bank provided the X-ray crystallography target, M^pro^, of SARS-CoV-2 (PDB code: 6LU7) [40]. Additionally, it was ready for docking by carefully following the previously detailed methods [41,42]. Furthermore, the downloaded protein was energy-reduced, 3D-hydrogen-loaded, and error-corrected [43,44]. The newly created molecules were substituted for the ligand location in a general docking approach. The co-crystallized ligand site was chosen as the docking site after adjusting the default program settings that were provided [45]. In a nutshell, the dummy atoms method was used to select the docking point. The placement and scoring procedures that were chosen were the triangle matcher and London dG. Out of a total of 100 poses for each docked molecule, the stiffer receptor was employed as the new refining strategy and the GBVI/WSA dG was employed as the new scoring methodology [46,47]. The optimal site for each ligand with the highest favorable scores, binding modes, and RMSD values was selected for further investigation. In the first step of the program validation method for the MOE program used, the co-crystallized instinctual inhibitor (N3) was redocked at its binding pocket of the generated main protease [48,49]. By obtaining a low root mean square deviation value (1.29) when comparing the freshly synthesized compounds and the redocked N3 ligand, a valid performance was demonstrated.

#### 2.2.1. Molecular Dynamics Simulation (MDs)

MD simulations were performed using the Desmond 2020.1 (Schrödinger, New York, NY. 2017) from Schrödinger, LLC on the docked complex for 6LU7 with the 8a ligand. In this system, the explicit solvent model with the TIP3P water molecules and the OPLS-2005 force field [50,51,52] were applied in a period boundary salvation box with dimensions of 10 Å x 10 Å x 10 Å [53]. Na^+^ ions were supplied to the system to balance the 0.15 M charge, and NaCl solutions were added to mimic physiological conditions. To retrain the system over the protein ligand complexes, the system was initially equilibrated using an NVT ensemble for 10 ns. Following the preceding phase, an NPT ensemble was used to complete a brief run of equilibration and minimization for 12 ns. The Nose–Hoover chain coupling approach [45,54] was used to set up the NPT ensemble and the variable temperature. Throughout all simulations, an active suspension of 1.0 ps and a pressure of 1 bar were maintained. The time step used was 2fs. The Martyna–Tuckerman–Klein chain coupling technique was used to manage pressure [55], employing a barostat method with a 2 ps relaxed period. The long-range electrostatic interactions were estimated using the particle mesh Ewald method [56], with the radius for the coulomb interactions set at 9. A RESPA integrator was used to determine the bonded forces for each trajectory during a time step of 2 fs. Making use of Geo Measures v0.8 ( https://github.com/lkagami/geo_measures_pymol, accessed on 1 January 2023) [57], the complexes underwent primary component analysis (PCA). Geo Measures is provided with a substantial library of g sham and eigen values, which are represented in a 3D visual using the Python program matplotlib (https://github.com/matplotlib/matplotlib, accessed on 1 January 2023). The final production run lasted 100 ns. The root mean square deviation (RMSD), radius of gyration (Rg), root mean square fluctuation (RMSF), quantity of hydrogen (H-bonds), salt bridges, and SASA were calculated to monitor the stability of the MD simulations.

#### 2.2.2. Binding Free Energy Analysis

The ligand–protein complex binding free energies were calculated using the molecular mechanics combined with the generalized Born surface area (MM-GBSA) method. Over the previous 50 frames, the Prime MM-GBSA binding free energy in the simulation trajectory with a one-step sampling size was calculated using the thermal mmgbsa.py Python script. The binding free energy of the Prime MM-GBSA (kcal/mol) was determined using the additivity concept, which required adding up each individual energy module, such as the columbic, covalent, hydrogen bond, van der Waals, self-contact, lipophilic, solvation of protein, and ligand modules.

The following equation is applied to determine Gbind:

(1) $$\Delta G_{bind}=\Delta G_{MM}+\Delta G_{Solv}-\Delta G_{SA}$$

In which:

- Δ*G_bind_* specifies the binding free energy;
- Δ*G_MM_* specifies the difference between the free energies of the ligand–macromolecule complex and the total energies of receptor and ligand in isolated forms;
- Δ*G_Solv_* specifies the differences in the GSA solvation energies of the ligand–macromolecule complex and the sum of the solvation energies of the receptor and the ligand in the unbound state;
- Δ*G_SA_* specifies the difference in the surface area energies for the receptor and the ligand.

## 3. Results and Discussion

### 3.1. Chemistry

**Scheme 1:**
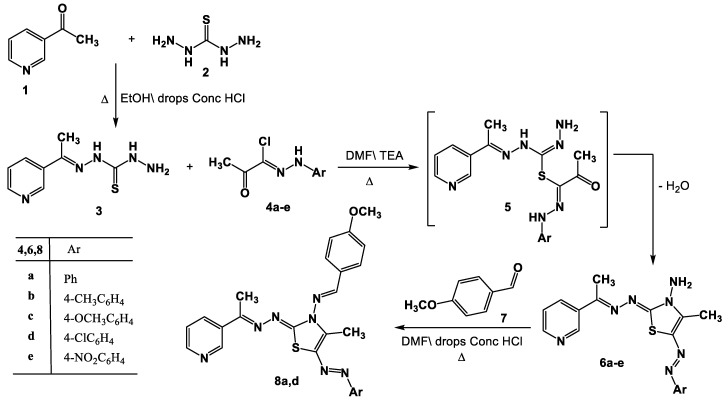
Synthesis of thiazoles **6a**–**e** and Schiff bases **8a,d**.

N’-(1-(Pyridin-3-yl)ethylidene)hydrazinecarbothiohydrazide (**3**) was prepared via the reaction of 3-acetylpyridine **1** with thiocarbohydrazide **2** in DMF in the presence of a catalytic amount of HCl under reflux in DMF (Scheme 1). Product **3** was elucidated based on spectral (IR, ^1^H-NMR, mass) and elemental data (see Experimental part).

The thiazole derivatives **6a**–**e** were produced through the reaction of compound **3** with the hydrazonoyl chlorides **4a**–**e** [58] in the presence of Et_3_N. This was achieved by first performing a substitution reaction with the removal of the HCl molecule to produce the substituted intermediate **5**, which was then followed by in situ cyclization with the removal of the water molecule (Scheme 1). Elemental analysis and spectral data (^1^H-NMR, mass, IR) were used to clarify the structure of the products **6a**–**e**. In each case, two stretching bands at 1692 and 3421–3160 cm^−1^, attributed to the carbonyl and NH groups, could be seen in the IR spectra of product **6**. The singlet signal at *δ* =10.69 ppm associated with the -NH proton was observed in the ^1^H-NMR spectra of compound **6**, in addition to the aromatic and alkyl protons. Each mass spectrum of products **6a**–**e** showed a molecular ion peak with the appropriate molecular weight for that molecule.

It was proposed that the hydrazine carbon of compound **4** is initially attacked by the thiol group of compound **3** to yield intermediate **5**, which is then cyclized to products **6**. By forming the Schiff bases **8a** and **8d** as a result of their interactions with 4-methoxybenzaldehyde **7** while being refluxed in acetic acid, the structural integrity of product **6** was further demonstrated. The structures of the isolated products **8a** and **8d** were elucidated based on their ^1^H-NMR, IR, and mass spectra (see experimental section).

**Scheme 2:**
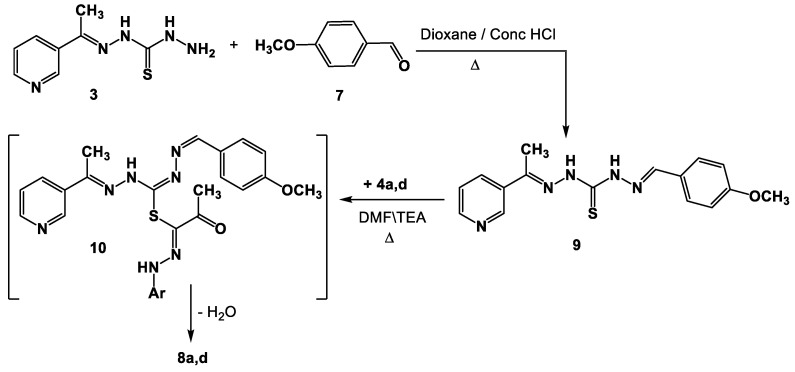
Alternative synthesis of Schiff bases derivatives **8a,d**.

A different synthetic approach might be used to create the real samples of **8a,d**. Thus, Schiff base **9** was produced as a result of compound **3** reacting with 4-methoxybenzaldehyde **7** while being heated in EtOH\AcOH. After reacting with hydrazonoyl halides **4a,d** in refluxing DMF, compound **9** produced the corresponding authentic samples **8a,d** by forming intermediate **10** (Scheme 2).

**Scheme 3:**
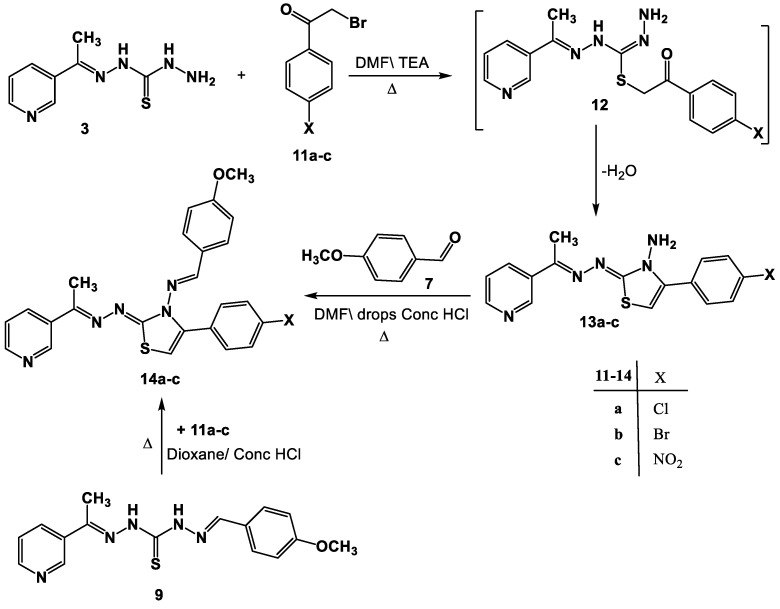
Synthesis of thiazoles **13a**–**c** and Schiff bases **14a**–**c**.

Through its reaction with α-bromoketones, the viability of compound **3** as a building block for the synthesis of additional groups of the anticipated biologically active thiazoles was also investigated. Therefore, just one isolable product was produced in each case when compound **3** was allowed to react with the substituted phenacyl bromides **11a**–**c** in DMF\TEA (Scheme 3). Based on the ^1^H-NMR, mass and IR spectral data, the latter products were determined to be **13a**–**c**. The ^1^H-NMR spectra of product **13a**, considered to be a sample example of the product **13**, revealed two singlet signals assigned to the NH_2_ and thiazole-H5 protons, in addition to the predicted signals assigned to the aromatic protons. The mass spectra of products **13a**–**c** demonstrated peaks that matched their molecular ions.

Moreover, N-aminothiazoles **13a**–**c** reacted with aldehyde **7** to yield the respective Schiff bases **14a**–**c** (Scheme 3). The structure of compound **14** was deduced from its spectral data (IR, ^1^H-NMR, and MS) and elemental analyses (*cf*. experimental part).

A different strategy was used to provide further support for the structure allocated to products **14a**–**c**. As a result, the reaction between **9** and **11a**–**c** in DMF under reflux produced a product that was identical to the product produced by the reaction between **13a**–**c** and **7** in all aspects (Scheme 3).

### 3.2. Physiochemical and Pharmacokinetics Profiling

**Figure 2:**
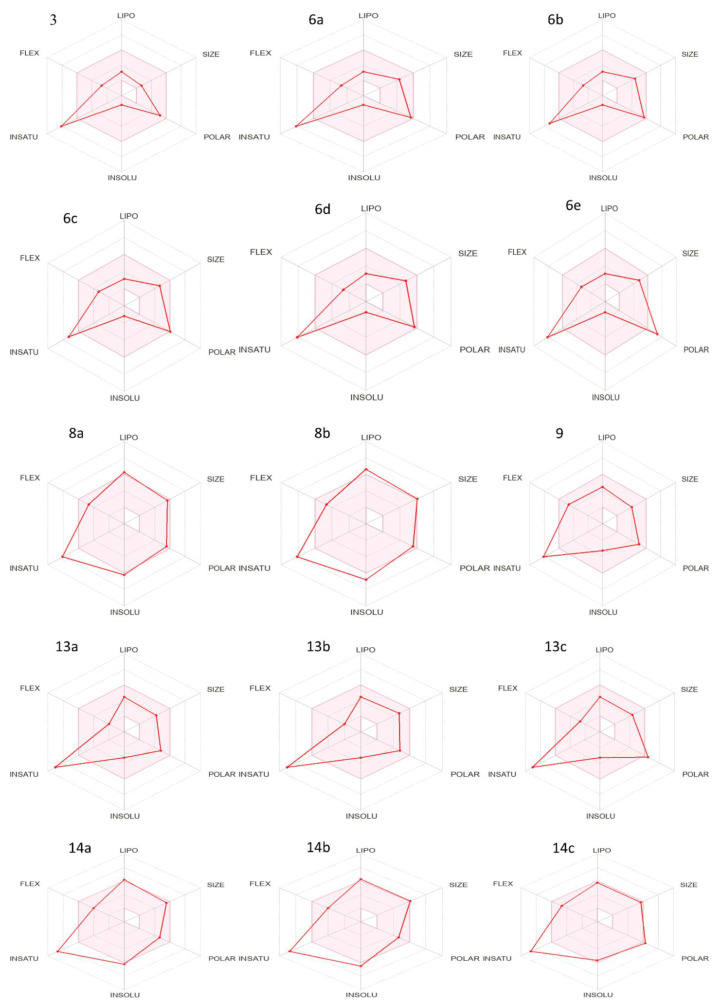
Bioavailability radar of newly synthesized compounds **3**–**14c**.

An ADMET investigation on all synthesized compounds was evaluated using the SwissADME platform (http://www.swissadme.ch/, last accessed on 21 December 2022). To ensure that the specified compounds were new drugs, the SwissADME tool was applied [59,60]. Table **1a–c** contain data of the physicochemical parameters. The listed data in Table 1a–c, show that all synthesized compounds except **8d** and **14b** have a molecular weight < 500 [61], which increased the transport and absorption as well as improved the transmissibility of these thiazole compounds to membranes. With the exception of 6e and **13c**, all of these thiazole derivatives’ topological polar surface areas (TPSA) were less than 140A^0^, according to the reported range (Table 1b,c) [62]. The lipophilicity (Log P) values of all the thiazole derivative examined lie within the 1.5–4.81 range, being acceptable as per Lipinski’s five-factor rule. It is important to note that lipophilicity is associated to toxicity [63]. The findings indicate that the physicochemical properties of the newly synthesized thiazole derivatives are within the acceptable range, as shown by the bioavailability radar (Figure 2).

A molecule’s druglikeness is represented by the bioavailability radar. The pink area corresponds to the optimal range for each property (lipophilicity: Log P between 1.52 and 4.81; size: MW between 209 and 504 g/mol; polarity: TPSA between 92 and 142 0A2; solubility: log S no higher than 6; etc.), with the pink area representing the best range for each property. Carbons must make up at least 0.25;2.4 of the sp3 hybridization’s carbon content to be considered saturated.

### 3.3. Molecular Docking Studies

**Figure 3:**
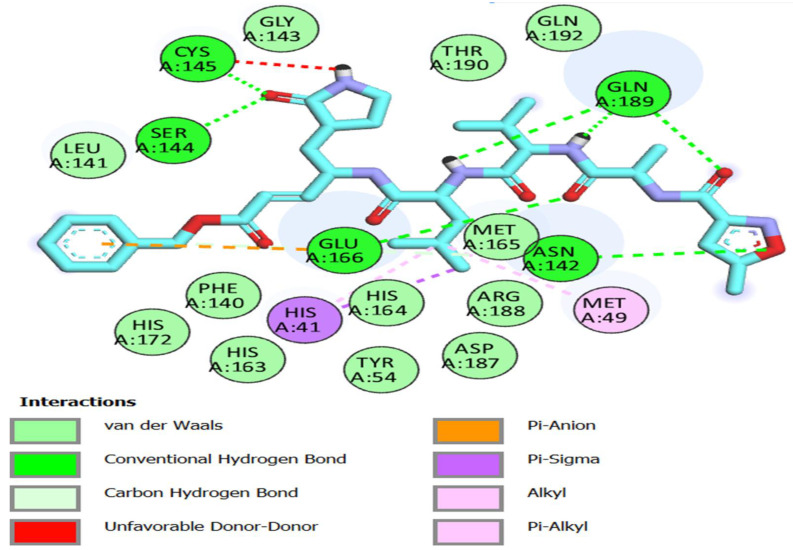
Two-dimensional interactions of re-docked co-crystalized Ligand (N3) onto the active site of M^pro^.

**Figure 4:**
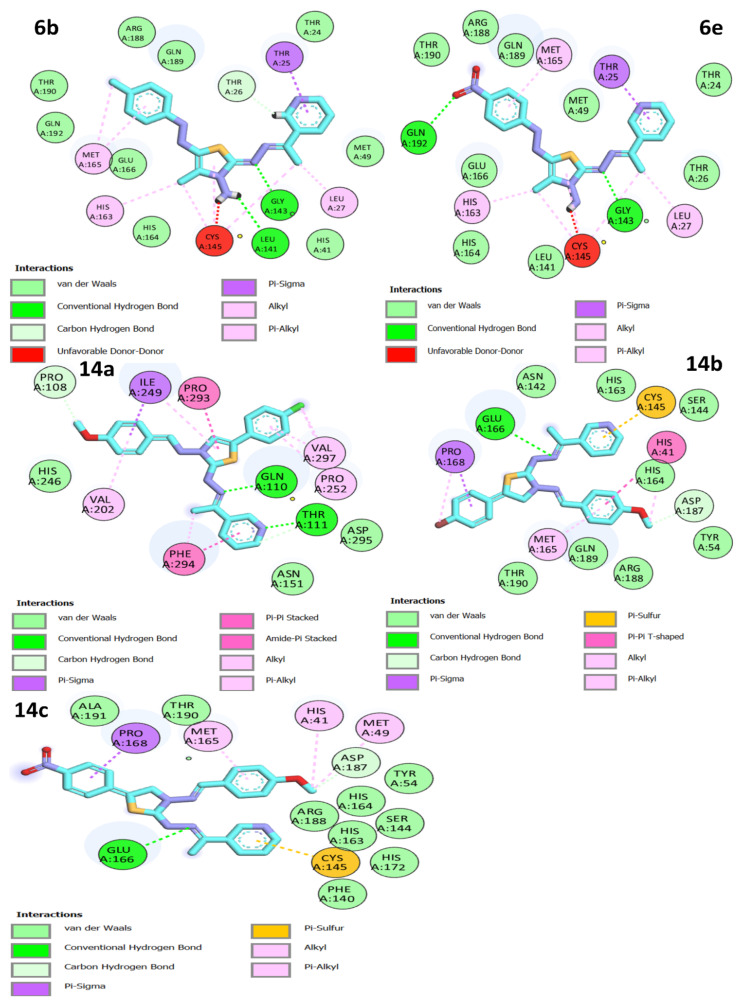
Two-dimensional interactions of docked compounds **6b, 6e, 14a, 14b**, and **14c** into the active site of M^pro^.

**Figure 5:**
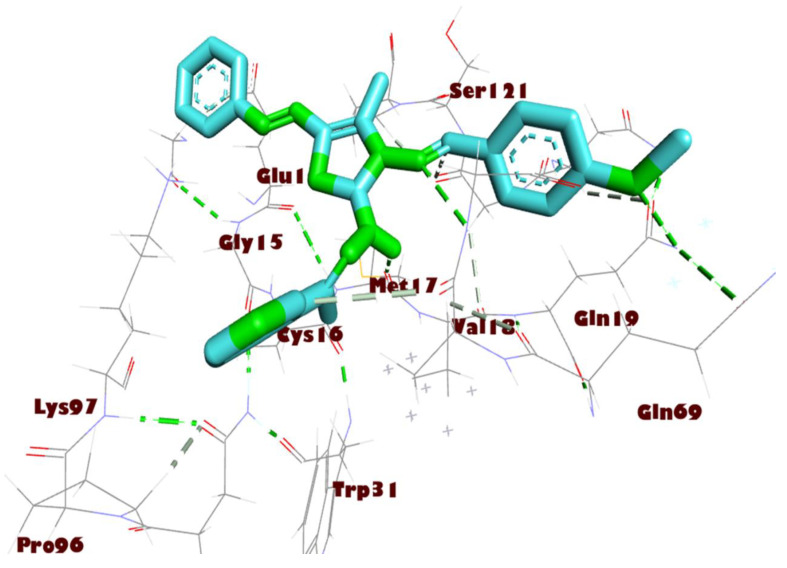
Interactions of compound **8a** with Mpro residues in three dimensions. Green dashed lines are used to indicate the hydrogen bonding.

**Figure 6:**
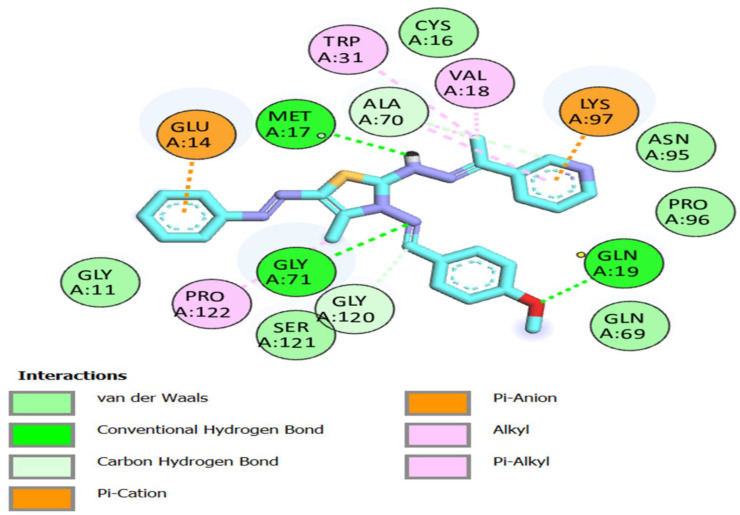
Compound **8a**: 2D interactions with Mpro residues.

**Figure 7:**
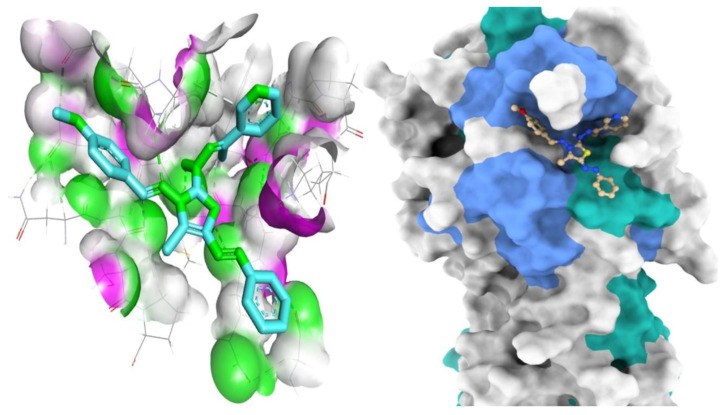
Mapping surface demonstrating compound **8a** existing in the Mpro active pocket.

Using a molecular docking simulation, the fifteen thiazole derivatives were tested for their capacity to engage with the main protease of COVID-19 (Pdb ID: 6LU7). Table 2 and Figure 3, Figure 4, Figure 5, Figure 6 and Figure 7 list the outcomes of this docking investigation. First, a redock was performed on the co-crystallized ligand (N3) for verification. ASN 142, GLY 143, GLU 166, GLN 189, SER 144, and CYS 145 residues formed an H-bond acceptor with the C=O of the co-crystallized ligand, demonstrating a high docking score of -8 kcal/mol (Figure 3).

All the thiazole compounds under investigation produced docking scores between −5.8 and −8.6 kcal/mol. The docking scores for the thiazole derivatives **6b**, **6e**, **14a**, **14b**, and **14c** were higher than those for the co-crystallized ligand. As is shown in Table 2 and Figure 4, these thiazole compounds were incorporated into the SARS CoV-2 main protease (Pdb: 6LU7) active site through interactions with the amino acid residues, which were H-acceptor, H-donor, and hydrophobic. Compound **8a** showed the highest binding affinity, demonstrating a binding energy value of −8.6 Kcal/mol against the active site of Mpro (Table 2). It showed three H-bonds with MET 17, GLN 19, and GLY 71, six hydrophobic interactions with GLU14, GLY 120, ALA 70, LYS 97, VAL 18, and TRP 31, as well as one electrostatic interaction with LYS 97 (Figure 4, Figure 5 and Figure 6). In light of these encouraging docking simulation results using the SARS CoV-2 main protease (Mpro) (Pdb ID: 6LU7), we recommend that thiazole derivatives be tested in vitro as antivirals to suppress the SARS CoV-2 main protease.

### 3.4. Simulation of Molecular Dynamics

**Figure 8:**
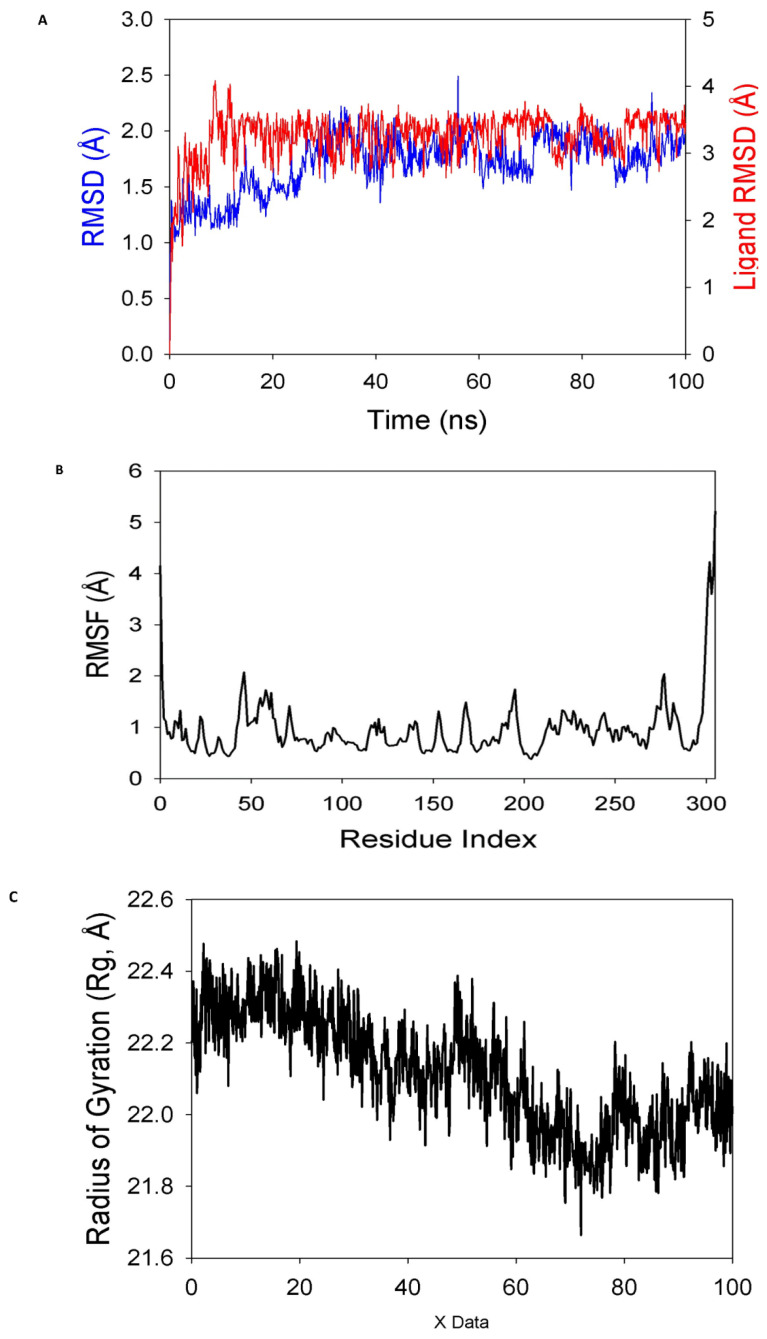

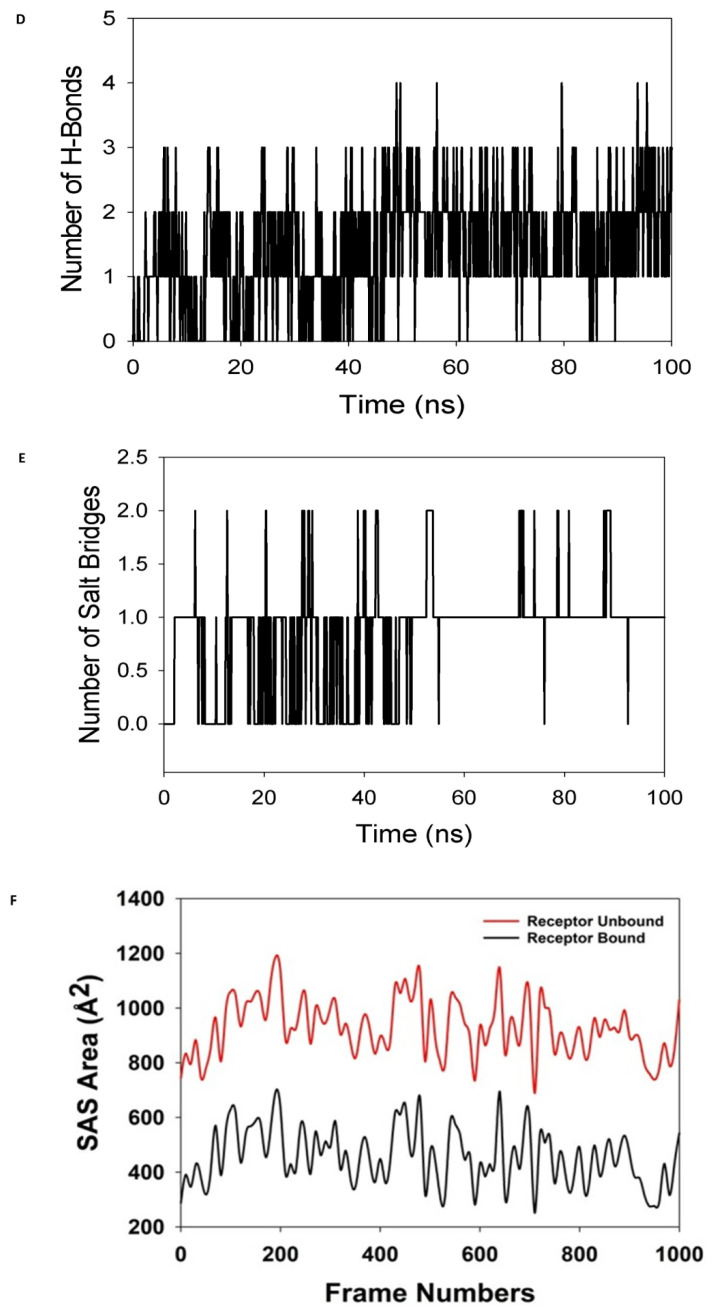
MD simulation analysis of 100 ns trajectories of (**A**) Cα backbone of (Mpro) (6LU7) + **8a** ligand, (**B**) RMSF of Cα backbone of 6LU7 bound with **8a**-ligand, (**C**) radius of gyration (Rg) of Cα backbone of 6LU7 bound with **8a,** (**D**) formation of hydrogen bonds in 6LU7 bound with **8a** complex, (**E**) numbers of salt bridge formation between and **8a**, and (**F**) solvent-accessible surface area of 6LU7 bound with **8a** complex.

The stability and convergence of the **8a**-ligand for the COVID-19 major protease (Mpro) (PDB ID: 6LU7) were investigated using molecular dynamics modelling (MD) techniques. The simulation of 100 ns demonstrated a steady conformation when comparing the findings of the root mean square deviation (RMSD). When it was coupled with the **8a**-ligand, the 6LU7 C-RMSD backbone produced a deviation of 1.9 A^0^. (Figure 8A), while the ligand RMSD of **8a** was depicted as 2.0 A^0^ (Figure 8A). Good convergence and stable conformations throughout the simulation are indicated by the stable RMSD graphs. The strong binding affinity of the ligand suggests that when **8a** is linked to 6LU7, it is quite stable in its complex. Although the root mean square fluctuations (RMSF) plot for the 6LU7 protein showed small fluctuation spikes, no substantial spikes were found, suggesting that the residues may be more flexible. Most of the residues showed minimal fluctuation over the duration of the 100 ns simulation (Figure 8B), which represents stable amino acid conformations. Since the protein structure is stiff during simulation in ligand-bound conformations, it can be deduced from RMSF plots.

The radius of gyration (Rg) measures a protein’s degree of compactness. This experiment saw a decrease in the radius of gyration (Rg) of the 6LU7 C-backbone linked to the **8a**-ligand from 22.3 to 22.01 (Figure 8C). When the gyration (Rg) is noticeably reduced, this indicates that the protein is strongly oriented in a ligand-bound state. The presence of hydrogen bonds between the protein and the ligand points to the stability and strong interaction of the complex. Throughout the 100 ns of the simulation, there were considerable amounts of hydrogen bonds between compound **8a** and 6LU7 (Figure 8D). The average constant number of hydrogen bonds between 6LU7 and **8a**-ligand was three on average (Figure 8D). Salt bridges were formed between the oppositely charged residues close to each other and played a significant role in protein stability [64]. In this study, average single numbers of salt bridges were formed between 6LU7 and the **8a**-ligand (Figure 8E). An Rg analysis was followed by similar patterns being observed in the solvent accessible surface area (SASA), both in the ligand-bound and unbound states. It is evident from (Figure 8F) that the protein 6LU7 had a high surface area, which was accessible to the solvent when the **8a**-ligand was not attached to the receptor (Figure 8F, red). When using the **8a**-ligand to bind, the SASA value decreased in comparison to the unbound state (Figure 8F, black). According to the overall analysis of the Rg, the matching proteins were compelled to become more compact and less flexible when the ligands were bound.

#### 3.4.1. Calculations of Molecular Mechanics Generalized Born Surface Area (MM-GBSA)

The binding free energy and additional contributing energy in the form of MM-GBSA were calculated for the 6LU7 + **8a** complex using the MD simulation trajectory. This was followed by Rg analysis, which likewise showed a similar trend. The results (Table 3) suggested that the maximum contribution to ΔG_bind_ in the stability of the simulated complexes were due to ΔG_bind_Coulomb, ΔG_bind_vdW, ΔG_bind_H_bond_, and ΔG_bind_Lipo, while ΔG_bind_Covalent and ΔG_bind_SolvGB were linked to the corresponding complexes’ instability. The 6LU7 + **8a** complex had comparatively higher binding free energies, higher than other complexes (Table 3). The potential for **8a** to bind to protein with a high affinity, efficiency, and the capacity to assemble a stable protein–ligand complex was substantiated by these findings.

#### 3.4.2. Principal Component Analysis

**Figure 9:**
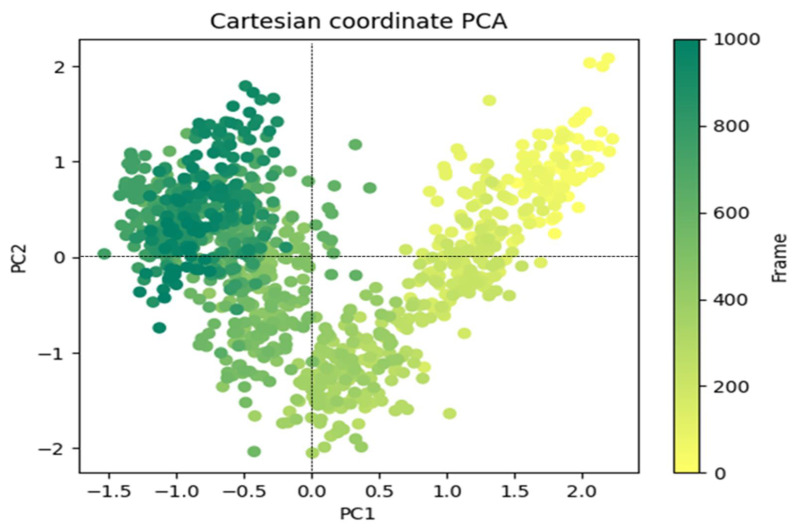
PCA analysis of Eigen values of 1000 frame Cartesian coordinates from the MD trajectory for 100 ns for 6LU7 + **8a**.

The outcomes of a study to explain the random, global mobility of the atoms in amino acid residues are displayed in Figure 9’s principal component analysis (PCA) of the MD simulation trajectories for 6LU7 + **8a**. The more flexible scattered trajectories (0–600 frames) are interpreted by this technique as a result of non-correlated global motion due to the protein structure’s randomness. A covariance matrix contained the internal coordinate mobility into three dimensions throughout the spatial time of 100 ns. Orthogonal sets, or eigenvectors, were used to represent the rational motion of each trajectory. The MD simulation trajectory of the Cα atoms of the 6LU7 + **8a** protein displayed more unordered orientation in PC1 and PC2 modes and was oriented more toward a negative correlation from the initial 600 frames (Figure 9). Interestingly, for the last 400 frames (from 600–1000), it exhibited a positive correlation motion and clustered into a more oriented manner. As a result, it was obvious that the centering of the frames in a single cluster by 6LU7 + **8a** (dark green) indicated that the periodic motion of MD trajectories was caused by steady, structural global motion. Consequently, the frames become more stable at the completion of the simulation (Figure 9).

## 4. Conclusions

According to the current work, a novel series of thiazole clubbed pyridines was created by reacting a pyridine thiocarbohydrazone derivative with a variety of hydrazonoyl halides and α-bromoacetophenones. The physicochemical parameters, toxicity assessment, and molecular docking approaches showed that compound (**8a**) was not toxic, did not violate Lipinski’s rule of five, and could fit well with the binding site of the SARS-CoV-2 target protease through interactions with essential amino acids, hence inhibiting the replication function of the virus. Furthermore, the compound (**8a**) complex with the SARS-CoV-2 target protease showed stability during the 100 ns trajectory of the molecular dynamic simulation analysis. The predicted findings could be a helpful suggestion for us and other scientists to conduct further confirmation via in vitro experimental studies. Nevertheless, the preliminary results of the present study may pave the way for developing more potent agents against SARS-CoV-2 in the near future.

## Figures and Tables

**Table 1:** (**a**). Physiochemical and pharmacokinetics profiling for compounds **3** and **6a**–**d**, (**b**) physiochemical and pharmacokinetics profiling for compounds **6e, 8a,d, 9,** and **13a**, and (**c**) physiochemical and pharmacokinetics profiling for compounds **13b,c** and **14a**–**c**.

a					
Properties	Compounds				
	3	6a	6b	6c	6d
Formula	C_8_H_11_N_5_S	C_17_H_17_N_7_S	C_18_H_19_N_7_S	C_18_H_19_N_7_OS	C_17_H_16_ClN_7_S
Molecular weight	209.07 g/mol	351.43 g/mol	365.46 g/mol	381.45 g/mol	385.87 g/mol
N. Heavy atoms	14	25	26	27	26
Number of aromatic heavy atoms	6	18	17	16	16
Number of Rotatable Bonds	4	4	4	5	4
HBA	5	5	5	6	5
HBD	4	1	1	1	1
Molar Refractivity	58.6	100.24	105.20	106.73	105.25
TPSA	107.42 Å^2^	121.52 Å^2^	121.52 Å^2^	130.75 Å^2^	121.52 Å^2^
Log P	1.52	1.52	1.52	1.52	1.52
Log S	−1.82 Very soluble	−1.20 Very soluble	−1.20 Very soluble	−1.20 Very soluble	−1.20 Very soluble
(GI absorption)	High	High	High	High	High
BBB	Nil	Nil	Nil	No	No
CYP1A2, CYP2C19, CYP2C9, CYP2D6, CYP3A4	No	No	No	No	No
	No	No	No	No	No
	No	No	No	No	No
	No	No	No	No	No
	No	No	No	No	No
Druglikeness (Lipinski)	Yes 0 violations	Yes 0 violations	Yes 0 violations	Yes 0 violations	Yes 0 violations
**b**					
**Properties**	**Compounds**				
	**6e**	**8a**	**8d**	**9**	**13a**
Formula	C_17_H_16_N_8_O_2_S	C_25_H_23_N_7_OS	C_25_H_22_ClN_7_OS	C_16_H_17_N_5_OS	C_16_H_14_ClN_5_S
Molecular weight	396.43 g/mol	469.56 g/mol	504.01 g/mol	327.40 g/mol	343.83 g/mol
Number of aromatic heavy atoms	28	34	35	23	23
Number of Rotatable Bonds	17	23	23	12	17
HBA	5	7	7	7	3
HBD	7	7	7	4	3
Molar Refractivity	1	0	0	2	1
TPSA	109.06	136.02	141.03	95.37	95.10
Log P	167.34 Å^2^	117.09 Å^2^	117.09 Å^2^	102.99 Å^2^	96.80 Å^2^
Log S	1.52	4.81	4.95	2.67	2.67
(GI absorption)	−1.20 Very soluble	−6.24 Poorly soluble	−6.83 Poorly soluble	−3.28 Moderately soluble	−3.28 Moderately soluble
BBB	High	Low	Low	High	High
Number of aromatic heavy atoms	No	No	No	No	No
CYP1A2, CYP2C19, CYP2C9, CYP2D6, CYP3A4	No	No	No	No	No
	No	No	No	No	No
	No	No	Yes	Yes	Yes
	No	No	No	No	No
	No	No	No	Yes	Yes
Druglikeness (Lipinski)	Yes 0 violations	Yes 0 violations	Yes 1 violation: MW > 500	Yes 0 violations	Yes 0 violations
**c**					
**Properties**	**Compounds**				
	**13b**	**13c**	**14a**	**14b**	**14c**
Formula	C_16_H_14_BrN_5_S	C_16_H_14_N_6_O_2_S	C_24_H_20_ClN_5_OS	C_24_H_20_BrN_5_OS	C_24_H_20_N_6_O_3_S
Molecular weight	388.28 g/mol	354.39 g/mol	461.97 g/mol	506.42 g/mol	472.52 g/mol
N. Heavy atoms	23	25	32	32	34
Number of aromatic heavy atoms	17	17	23	23	23
Number of Rotatable Bonds	3	4	6	6	7
HBA	3	5	5	5	7
HBD	1	1	0	0	0
Molar Refractivity	97.79	98.91	130.88	133.57	134.69
TPSA	96.80 Å^2^	142.62 Å^2^	92.37 Å^2^	92.37 Å^2^	138.19 Å^2^
Log P	2.67	2.67	4.40	4.46	3.66
Log S	−3.28 Moderately soluble	−3.28 Moderately soluble	−6.22 Poorly soluble	−6.54 Poorly soluble	−5.69 Moderately soluble
(GI absorption)	High	High	High	High	Low
BBB	No	No	No	No	No
CYP1A2, CYP2C19, CYP2C9, CYP2D6, CYP3A4	No	No	No	No	No
	No	No	Yes	Yes	Yes
	Yes	Yes	Yes	Yes	Yes
	No	No	No	No	No
	Yes	Yes	Yes	No	Yes
Druglikeness (Lipinski)	Yes 0 violations	Yes 0 violations	Yes 0 violations	Yes 1 violation: MW > 500	Yes 0 violations

**Table 2:** The redocked N3 inhibitor within the active site of the SARS CoV-2 main protease (6LU7), together with the interactions and binding scores of the substances that were tested.

Compounds	Binding Energy (kcal/mol)	Hydrogen Bond Interactions	Distance (Å)	Hydrophobic Interactions	Distance (Å)
**3**	−5.8	GLN 110 THR 111 SER 158 THR 292 ASP 295	2.75 2.25 2.32 2.16 2.49	VAL 104 ILE 106 GLN 110	3.82 3.93 3.67
**6a**	−7.8	GLY 143 SER 144 CYS 145	2.07 2.29 2.72	THR 25 ASN 142 MET 165 GLN 189	3.85 3.61 3.43 3.35
**6b**	−8.3	LEU 141 GLY 143 SER 144 CYS 145	1.91 1.78 2.65 2.92	THR 25 LEU 27 MET 165 GLN 189	3.94 3.80 3.32 3.54
**6c**	−7.9	GLY 143 SER 144 CYS145 HIS 164 THR 190	1.66 3.29 3.42 4.10 3.96	THR 25 LEU 27 MET 165 GLN 189	3.98 3.95 3.34 3.38
**6d**	−7.3	LEU 141 GLY 143 SER 144 CYS 145	2.42 1.63 2.51 2.79	THR 25 LEU 27 MET 165 GLN 189	3.82 3.97 3.42 3.38
**6e**	−8.4	GLN 192 GLY 143 SER 144 CYS 145	2.35 1.72 2.61 2.92	LEU 27 MET 165 HIS 163	3.90 3.41 3.43
**8a**	−8.6	GLY 71 GLN 19 MET 17	3.41 2.99 3.52	GLU 14 GLY 120 ALA 70 LYS 97 VAL 18 TRP 31	2.67 3.91 3.32 3.78 2.98 3.53
**8d**	−7.8	GLY 143 GLU 166	3.47 2.76, 2.86	MET 165	3.91
**9**	−7.2	MET 17 GLN 19 GLY 71	2.58 2.28 2.48	VAL 18 GLN 19 TRP 31 GLN 69 PRO 96	3.69 3.72 3.68, 3.73 3.73 3.99
**13a**	−6.8	GLU 14 GLY 15	3.14 2.24,3.65	ALA 70 VAL 73 PRO 96	3.38 3.94 3.55
**13b**	−7.2	GLY 15	2.5	GLU 14 ALA 70 PRO 96	3.92 3.46 3.55
**13c**	−6.7	GLU 166	3.36	PRO 168 GLN 189	3.74 3.56
**14a**	−8.4	GLN 110 THR 111	2.39	PHE 294 VAL 202 PRO 252 PRO 292 ILE 249	3.64 3.68 3.72, 3.65 3.45 3.49
**14b**	−8.3	GLU 166	2.86	MET 165 GLU 166 PRO 168 GLN 189	3.76 3.90 3.73 3.74
**14c**	−8.1	GLU 166	2.32	MET 165 GLN 189 PRO 168 CYS 145	3.53 3.48 3.44 3.84
**Inhibitor N3**	−8	ASN 142 GLY 143 GLU 166 GLN 189 SER 144 CYS 145	2.81 2.38 3.27 2.01,2.72,2.58 2.67 2.72	HIS 41 MET 49 ASP 187 GLN 189 HIS 163 HIS 172	3.68 3.64 3.83 3.69 5.02 5.40

**Table 3:** Components of the binding free energy for the 6LU7 + **8a** as determined by MM-GBSA.

Energies (kcal/mol)	6LU7 + 8a
ΔG_bind_	−56.81 ± 6.79
ΔG_bind_Lipo	−18.08 ± 1.04
ΔG_bind_vdW	−48.49 ± 2.18
ΔG_bind_Coulomb	−25.47 ± 6.20
ΔG_bind_H_bond_	−1.73 ± 0.34
ΔG_bind_SolvGB	31.74 ± 3.34
ΔG_bind_Covalent	5.23 ± 4.41

## Data Availability

The data presented in this study are available on request.

## Supplementary Materials

The following supporting information can be downloaded at: https://www.mdpi.com/article/10.3390/cimb45020093/s1, Physical and spectral data of the synthesized compounds and their ^1^H- and ^13^C-NMR spectra.
