# Supplementary material for: Synthesis, Molecular Docking, and Dynamic Simulation Targeting Main Protease (Mpro) of New, Thiazole Clubbed Pyridine Scaffolds as Potential COVID-19 Inhibitors
Source: Curr Issues Mol Biol. 2023 Feb 7;45(2):1422–42. doi: 10.3390/cimb45020093 (PMC9955078; doi:10.3390/cimb45020093)
Supplement: Supplementary file 1 [file cimb-45-00093-s001.zip › cimb-2179602-supplementary.pdf]

Supplementary File

Synthesis, Molecular Docking, and Dynamic Simulation Targeting Main Protease (Mpro) of New, Thiazole Clubbed Pyridine Scaffolds as Potential COVID-19 Inhibitors

Adel Alghamdi [1], Amr S. Abouzied [2,3], Abdulwahab Alamri [4], Sirajudheen Anwar [4], Mukhtar Ansari [5], Ibrahim Khadra [6], Yasser H. Zaki [7] and Sobhi M. Gomha [8,9,*]

1 Pharmaceutical Chemistry Department, Faculty of Clinical Pharmacy, Al Baha University, Al Baha P.O. Box 1988, Saudi Arabia
2 Department of Pharmaceutical Chemistry, College of Pharmacy, University of Hail, Hail 81442, Saudi Arabia
3 Department of Pharmaceutical Chemistry, National Organization for Drug Control and Research (NODCAR), Giza 12311, Egypt
4 Department of Pharmacology and Toxicology, College of Pharmacy, University of Hail, Hail 81442, Saudi Arabia
5 Department of Clinical Pharmacy, College of Pharmacy, University of Hail, Hail 81442, Saudi Arabia
6 Strathclyde Institute of Pharmacy and Biomedical Sciences, University of Strathclyde, 161 Cathedral Street, Glasgow G4 0RE, UK
7 Department of Chemistry, Faculty of Science, Beni-Suef University, Beni-Suef 62514, Egypt
8 Department of Chemistry, Faculty of Science, Islamic University of Madinah, Madinah 42351, Saudi Arabia
9 Department of Chemistry, Faculty of Science, Cairo University, Giza 12613, Egypt
* Correspondence: smgomha@iu.edu.sa or s.m.gomha@cu.edu.eg

3. Experimental

3.1. Chemistry

All melting points were determined on an electrothermal apparatus and were left uncorrected. Elemental analyses were carried out at the Microanalytical Center of Cairo University. Mass spectra were recorded on a GC-MS QP1000 EX Shimadzu. ^1H-NMR and ^{13}C-NMR spectra were recorded in DMSO solutions on BRUKER 400 FT-NMR system spectrometer and chemical shifts were expressed in ppm units using TMS as an internal reference. IR spectra were recorded (KBr discs) on a Shimadzu FT-IR 8201 PC spectrophotometer.

Synthesis of *N'*-(1-(pyridin-3-yl)ethylidene)hydrazinecarbothiohydrazide (3).

A mixture of 3-acetylpyridine (**1**) (1.21 g, 10 mmol) and thiocarbohydrazide (**2**) (1.06 g, 10 mmol) in 50 mL of EtOH was treated with catalytic quantities of concentrated HCl. For three hours, the reaction mixture was refluxed. To obtain the pure product of compound **3**, the precipitate that developed after cooling was filtered, washed with ethanol, and recrystallized from EtOH DMF as yellowish-white solid in 76% yield; m.p. 188-190 °C; IR (KBr): *v* 3427, 3349, 3228 (NH_2 and 2NH), 1604 (C=N) cm^{-1}; ^1H-NMR (DMSO-d_6): δ = 2.46 (s, 3H, CH_3), 3.25 (s, 2H, NH2), 7.73 (t, 1H, Pyr-H5), 8.06 (s, 1H, NH), 8.26 (d, 1H, Pyr-H4), 8.59 (d, 1H, Pyr-H6), 9.04 (s, 1H, Pyr-H2), 11.15 (s, 1H, NH) ppm; MS m/z (%): 209 (M^+, 46). Anal. Calcd: for $C_8H_{11}N_5S$ (209.07): C, 45.92; H, 5.30; N, 33.47. Found: C, 45.75; H, 5.24; N, 33.28%.

Synthesis of thiazole derivatives 6a-e and 13a-c

A catalytic amounts of TEA were added into a solution of N'-(1-(pyridin-3-yl)ethylidene)hydrazine- carbothiohydrazide (**3**) (0.209 g, 1 mmol) and the appropriate hydrazonoyl chlorides **4a-e** or α-bromoketones **11a-c** (1 mmol for each) in DMF (20 mL), and the reaction mixture was refluxed for 3-6 hours (monitored by TLC). Finally, the formed precipitate was isolated and recrystallized from the proper solvent to give products **6a–e** or **13a-c,** respectively. Below is a list of the isolated products' spectrum information and physical characteristics:

4-Methyl-5-phenyldiazenyl-2-((1-(pyridin-3-yl)ethylidene)hydrazineylidene)thiazol-3(2H)-amine (6a). Red solid, 78% yield, m.p. 155-157 ºC (EtOH); IR (KBr): *v* 3426, 3271 (NH_2), 1606 (C=N) cm^{-1}; ^1H-NMR (DMSO-d_6): δ = 2.38 (s, 3H, CH_3), 2.63 (s, 3H, CH_3), 5.81 (s, 2H, NH2), 7.18-7.66 (m, 6H, Ar-H and Pyr-H5), 8.20 (d, 1H, Pyr-H4), 8.58 (d, 1H, Pyr-H6), 9.02 (s, 1H, Pyr-H2) ppm; ^{13}C-NMR (DMSO-d_6): δ = 12.48, 14.14 (CH_3), 101.16, 119.08, 123.68, 129.26, 129.32, 129.51, 133.69, 133.77, 133.85, 134.42,

148.41, 150.19, 155.82 (Ar-C and C=N)ppm; MS *m/z* (%): 351 (M^+, 58). Anal. Calcd for $C_{17}H_{17}N_7S$ (351.13): C, 58.10; H, 4.88; N, 27.90. Found: C, 58.03; H, 4.66; N, 27.79%.

4-Methyl-2-((1-(pyridin-3-yl)ethylidene)hydrazineylidene)-5-((p-tolyldiazenyl)thiazol-3(2H)-amine (6b). Red solid, 77% yield, m.p. 172-174 ºC (EtOH); IR (KBr): *v* 3410, 3247 (NH_2), 1603 (C=N) cm^{-1}; ^{1}H-NMR (DMSO-d_6): δ = 2.20 (s, 3H, CH_3), 2.31 (s, 3H, CH_3), 2.61 (s, 3H, CH_3), 5.82 (s, 2H, NH2), 6.92-7.56 (m, 5H, Ar-H and Pyr-H5), 8.20 (d, 1H, Pyr-H4), 8.57 (d, 1H, Pyr-H6), 9.06 (s, 1H, Pyr-H2) ppm; MS *m/z* (%): 365 (M^+, 62). Anal. Calcd for $C_{18}H_{19}N_7S$ (365.14): C, 59.16; H, 5.24; N, 26.83. Found: C, 59.06; H, 5.41; N, 26.69%.

5-((4-Methoxyphenyl)diazenyl)-4-methyl-2-((1-(pyridin-3-yl)ethylidene)hydrazineylidene)thiazol-3(2H)-amine (6c). Dark red solid, 78% yield, m.p. 180-182 ºC (DMF); IR (KBr): *v* 3436, 3247 (NH_2), 1607 (C=N) cm^{-1}; ^{1}H-NMR (DMSO-d_6): δ = 2.38 (s, 3H, CH_3), 2.60 (s, 3H, CH_3), 3.78 (s, 3H, OCH_3), 5.74 (s, 2H, NH2), 6.99-7.65 (m, 5H, Ar-H and Pyr-H5), 8.19 (d, 1H, Pyr-H4), 8.57 (d, 1H, Pyr-H6), 9.01 (s, 1H, Pyr-H2) ppm; ^{13}C-NMR (DMSO-d_6): δ = 12.40, 14.77 (CH_3), 56.02, (OCH_3), 100.11, 115.06, 123.68, 124.04, 133.87, 134.10, 146.81, 146.92, 147.94, 150.54, 156.68, 160.66, 165.13 (Ar-C and C=N) ppm; MS *m/z* (%): 381 (M^+, 100). Anal. Calcd for $C_{18}H_{19}N_7OS$ (381.14): C, 56.68; H, 5.02; N, 25.70. Found: C, 56.57; H, 5.00; N, 25.57%.

5-((4-Chlorophenyl)diazenyl)-4-methyl-2-((1-(pyridin-3-yl)ethylidene)hydrazineylidene)thiazol-3(2H)-amine (6d). Orange solid, 78% yield, m.p. 187-189 ºC (EtOH \ DMF); IR (KBr): *v* 3427, 3244 (NH_2), 1613 (C=N) cm^{-1}; ^{1}H-NMR (DMSO-d_6): δ = 2.38 (s, 3H, CH_3), 2.62 (s, 3H, CH_3), 5.83 (s, 2H, NH2), 7.26-7.66 (m, 5H, Ar-H and Pyr-H5), 8.19 (d, 1H, Pyr-H4), 8.57 (d, 1H, Pyr-H6), 9.01 (s, 1H, Pyr-H2) ppm; MS *m/z* (%): 387 (M^++ 2, 30), 385 (M^+, 85). Anal. Calcd for $C_{17}H_{16}ClN_7S$ (385.09): C, 52.92; H, 4.18; N, 25.41. Found: C, 52.88; H, 4.04; N, 25.30%.

4-Methyl-5-((4-nitrophenyl)diazenyl)-2-((1-(pyridin-3-yl)ethylidene)hydrazineylidene)thiazol-3(2H)-amine (6e). Yellow solid, 77% yield, m.p. 204-206 ºC (EtOH \ DMF); IR (KBr): *v* 3451, 3254 (NH_2), 1614 (C=N) cm^{-1}; ^{1}H-NMR (DMSO-d_6): δ = 2.28 (s, 3H, CH_3), 2.47 (s, 3H, CH_3), 5.88 (s, 2H, NH2), 7.21-8.19 (m, 5H, Ar-H and Pyr-H5), 8.37 (d, 1H, Pyr-H4), 8.62 (d, 1H, Pyr-H6), 9.06 (s, 1H, Pyr-H2) ppm; MS *m/z* (%): 396 (M^+, 100). Anal. Calcd for $C_{17}H_{16}N_8O_2S$ (396.11): C, 51.51; H, 4.07; N, 28.27. Found: C, 51.40; H, 4.01; N, 28.14%.

4-(4-Chlorophenyl)-2-((1-(pyridin-3-yl)ethylidene)hydrazineylidene)thiazol-3(2H)-amine (13a). Yellow crystals, 79% yield, m.p. 188-190 ºC (EtOH); IR (KBr): *v* 3401, 3237 (NH_2), 1609 (C=N) cm^{-1}; ^{1}H-NMR (DMSO-d_6): δ = 2.44 (s, 3H, CH_3), 4.95 (s, 2H, NH2), 7.48-8.20 (m, 8H, Ar-H), 9.03 (s, 1H, Pyr-H2) ppm; ^{13}C-NMR (DMSO-d_6): δ = 14.26 (CH_3), 109.92, 120.02, 123.75, 130.29, 132.24, 137.77,

140.27, 146.10, 142.35, 149.37, 150.05, 161.72, 164.39 (Ar-C and C=N) ppm; MS *m/z* (%): 345 (M^++ 2, 24), 343 (M^+, 100). Anal. Calcd for $C_{16}H_{14}ClN_5S$ (343.07): C, 55.89; H, 4.10; N, 20.37. Found: C, 55.72; H, 4.03; N, 20.18%.

4-(4-Bromophenyl)-2-((1-(pyridin-3-yl)ethylidene)hydrazineylidene)thiazol-3(2H)-amine

(13b). Yellow solid, 79% yield, m.p. 202-204 ºC (DMF); IR (KBr): *ν* 3426, 3230 (NH_2), 1604 (C=N) cm^{-1}; ^{1}H-NMR (DMSO-d_6): δ = 2.27 (s, 3H, CH_3), 4.93 (s, 2H, NH2), 7.35-7.91 (m, 8H, Ar-H), 8.65 (s, 1H, Pyr-H2) ppm; MS *m/z* (%): 389 (M^++ 2, 22), 387 (M^+, 25). Anal. Calcd for $C_{16}H_{14}BrN_5S$ (387.02): C, 49.49; H, 3.63; N, 18.04. Found: C, 49.37; H, 3.51; N, 17.93%.

4-(4-Nitrophenyl)-2-((1-(pyridin-3-yl)ethylidene)hydrazineylidene)thiazol-3(2H)-amine

(13c). Brown solid, 85% yield, m.p. 214-216 ºC (DMF); IR (KBr): *ν* 3451, 3269 (NH_2), 1616 (C=N) cm^{-1}; ^{1}H-NMR (DMSO-d_6): δ = 2.36 (s, 3H, CH_3), 5.03 (s, 2H, NH2), 7.20 (s, 1H, Thiazole-H5), 7.73-8.29 (m, 5H, Ar-H), 8.38 (d, 1H, Pyr-H4), 8.79 (d, 1H, Pyr-H6), 9.09 (s, 1H, Pyr-H2) ppm; MS *m/z* (%): 354 (M^+, 51). Anal. Calcd for $C_{16}H_{14}N_6O_2S$ (354.09): C, 54.23; H, 3.98; N, 23.71. Found: C, 54.06; H, 3.74; N, 23.55%.

Synthesis of Schiff bases 8a,d and 14a-c.

A catalytic amounts of Conc HCl were added into a solution of 4-methoxybenzaldehyde (**7**) (1.36 g, 10 mmol) and the appropriate **8a,d** or **13a-c** (1 mmol for each) in DMF (20 mL), and the reaction mixture was refluxed for 2-4 hours (monitored by TLC). Finally, the formed precipitate was recrystallized from the proper solvent to give products **6a–e** or **13a-c,** respectively. Below is a list of the isolated products' spectrum information and physical characteristics:

1-(4-Methoxyphenyl)-N-(4-methyl-5-(phenyldiazenyl)-2-((1-(pyridin-3-yl)ethylidene)hydrazineylidene)thiazol-3(2H)-yl)methanimine (8a). Yellow solid, 82% yield, m.p. 207-209 ºC (DMF); IR (KBr): *ν* 3031, 2927 (CH), 1602 (C=N) cm^{-1}; ^{1}H-NMR (DMSO-d_6): δ = 2.42 (s, 3H, CH_3), 2.75 (s, 3H, CH_3), 3.79 (s, 3H, OCH_3), 7.03-7.75 (m, 11H, Ar-H, Pyr-H5 and Pyr-H4), 8.82 (d, 1H, Pyr-H6), 8.82 (s, 1H, Pyr-H2), 9.96 (s, 1H, CH=N) ppm; ^{13}C-NMR (DMSO-d_6): δ = 13.82, 14.59 (CH_3), 55.98 (OCH_3), 111.89, 119.42, 122.64, 123.75, 123.98, 133.35, 133.75, 135.31, 138.78, 141.27, 146.00, 147.35, 147.78, 148.36, 149.63, 150.25, 155.92, 162.26 (Ar-C and C=N) ppm; MS *m/z* (%): 469 (M^+, 85). Anal. Calcd for $C_{25}H_{23}N_7OS$ (469.17): C, 63.95; H, 4.94; N, 20.88. Found: C, 63.72; H, 4.84; N, 20.70%.

N-5-((4-chlorophenyl)diazenyl)-4-methyl-2-((1-(pyridin-3-yl)ethylidene)hydrazineylidene)thiazol-3(2H)-yl)-1-(4-methoxyphenyl)methanimine (8b). Red solid, 85% yield, m.p. 191-193 ºC (EtOH \ DMF); IR (KBr): *ν* 3047, 2938 (CH), 1605 (C=N) cm^{-1}; ^{1}H-NMR (DMSO-d_6): δ = 2.34 (s, 3H, CH_3), 2.47 (s, 3H,

CH_3), 3.79 (s, 3H, OCH_3), 6.98-7.81 (m, 10H, Ar-H and Pyr-H5), 8.48 (d, 1H, Pyr-H4), 8.61 (d, 1H, Pyr-H6), 9.05 (s, 1H, Pyr-H2), 9.93 (s, 1H, CH=N) ppm; MS *m/z* (%): 505 (M^++ 2, 24), 503 (M^+, 73). Anal. Calcd for $C_{25}H_{22}ClN_7OS$ (503.13): C, 59.58; H, 4.40; N, 19.45. Found: C, 59.43; H, 4.48; N, 19.29%.

N-(4-(4-Chlorophenyl)-2-((1-(pyridin-3-yl)ethylidene)hydrazineylidene)thiazol-3(2H)-yl)-1-(4-methoxyphenyl)methanimine (14a). Yellow crystals, 84% yield, m.p. 227-229 ºC (DMF); IR (KBr): *v* 3029, 2935 (CH), 1606 (C=N) cm^{-1}; ^{1}H-NMR (DMSO-d_6): δ = 2.46 (s, 3H, CH_3), 3.79 (s, 3H, OCH_3), 6.90-7.68 (m, 10H, Ar-H and Pyr-H5), 8.24 (d, 1H, Pyr-H4), 8.35 (d, 1H, Pyr-H6), 9.29 (s, 1H, Pyr-H2), 9.82 (s, 1H, CH=N) ppm; MS *m/z* (%): 348 (M^++ 2, 10), 461 (M^+, 33). Anal. Calcd for $C_{24}H_{20}ClN_5OS$ (461.11): C, 62.40; H, 4.36; N, 15. Found: C, 62.25; H, 4.24; N, 15.07%.

N-(4-(4-Bromophenyl)-2-((1-(pyridin-3-yl)ethylidene)hydrazineylidene)thiazol-3(2H)-yl)-1-(4-methoxyphenyl)methanimine (14b). Yellow crystals, 83% yield, m.p. 219-221 ºC (DMF); IR (KBr): *v* 3041, 2924 (CH), 1602 (C=N) cm^{-1}; ^{1}H-NMR (DMSO-d_6): δ = 2.36 (s, 3H, CH_3), 3.77 (s, 3H, OCH_3), 6.98-7.84 (m, 10H, Ar-H and Pyr-H5), 8.00 (d, 1H, Pyr-H4), 8.34 (d, 1H, Pyr-H6), 8.80 (s, 1H, Pyr-H2), 9.27 (s, 1H, CH=N) ppm; MS *m/z* (%): 507 (M^++ 2, 13), 505 (M^+, 15). Anal. Calcd for $C_{24}H_{20}BrN_5OS$ (505.06): C, 56.92; H, 3.98; N, 13.83. Found: C, 56.80; H, 3.73; N, 13.69%.

1-(4-Methoxyphenyl)-N-(4-(4-nitrophenyl)-2-((1-(pyridin-3-yl)ethylidene)hydrazineylidene)thiazol-3(2H)-yl)methanimine (14c). Yellow crystals, 83% yield, m.p. 219-221 ºC (DMF); IR (KBr): *v* 3049, 2929 (CH), 1605 (C=N) cm^{-1}; ^{1}H-NMR (DMSO-d_6): δ = 2.41 (s, 3H, CH_3), 3.78 (s, 3H, OCH_3), 6.98-7.78 (m, 10H, Ar-H and Pyr-H5), 8.21 (d, 1H, Pyr-H4), 8.36 (d, 1H, Pyr-H6), 9.35 (s, 1H, Pyr-H2), 9.95 (s, 1H, CH=N) ppm; MS *m/z* (%): 472 (M^+, 52). Anal. Calcd for $C_{24}H_{20}N_6O_3S$ (472.13): C, 61.01; H, 4.27; N, 17.79. Found: C, 60.89; H, 4.04; N, 17.57%.

Alternate synthesis for Schiff bases 8a,d and 14a-c

i. Synthesis of N'-(4-methoxybenzylidene)-2-(1-(pyridin-3-yl)ethylidene)hydrazine-1- carbothiohydrazide (9).

A catalytic amounts of concentrated HCl was added to a mixture of N'-(1-(pyridin-3-yl)ethylidene)hydrazine- carbothiohydrazide (**3**) (0.209 g, 1 mmol) and 4-methoxybenzaldehyde (**7**) (1.36 g, 10 mmol) in 30 mL of dioxane. The reaction mixture was refluxed for 3hr. The precipitate which formed was recrystallized from DMF to give pure product of compound **9** as yellowish-white solid in 79% yield; m.p. 227-229 °C; IR (KBr): *v* 3359, 3226 (NH), 1608 (C=N) cm^{-1}; ^{1}H-NMR (DMSO-d_6): δ = 2.37 (s, 3H, CH_3), 3.78 (s, 3H, OCH_3), 6.97-7.78 (m, 5H, Ar-H and Pyr-H5), 8.05 (d, 1H, Pyr-H4), 8.50 (d, 1H, Pyr-H6), 8.59 (s, 1H, Pyr-H2), 9.34 (s, 1H, CH=N), 11.37 (s, 1H, NH), 11.75 (s, 1H, NH) ppm; ^{13}C-NMR (DMSO-d_6): δ = 14.27 (CH_3), 56.03 (OCH_3), 116.27, 121.38, 123.00, 129.41, 131.06, 135.64, 138.96, 141.91,

147.00, 149.37, 155.29, 163.37 (Ar-C and C=N), 185.03 (C=S) ppm; MS m/z (%): 327 (M^+, 100). Anal. Calcd: for $C_{16}H_{17}N_5OS$ (327.12): C, 58.70; H, 5.23; N, 21.39. Found: C, 58.58; H, 5.06; N, 21.18%.

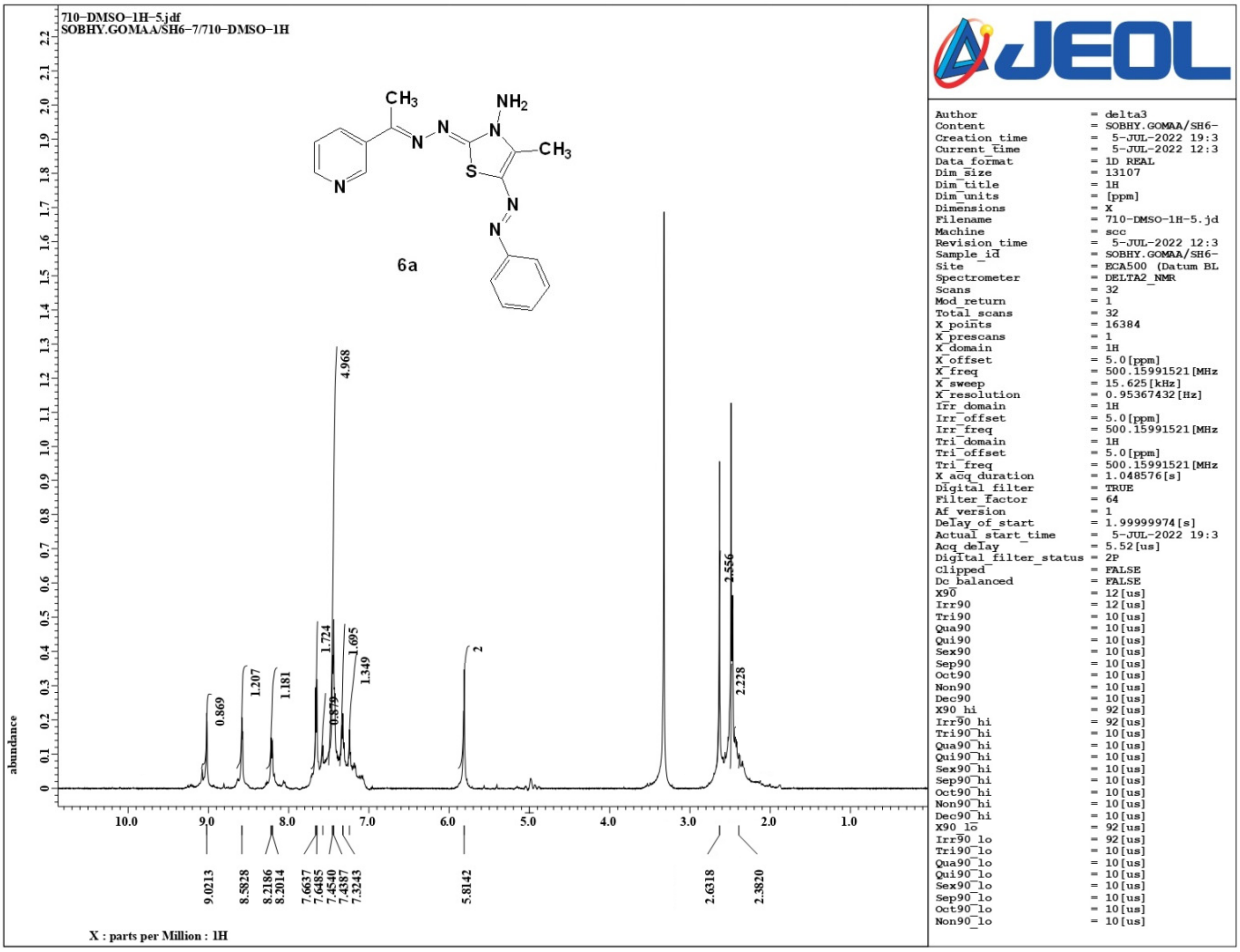

^{1}H-NMR spectra of compound **6a**

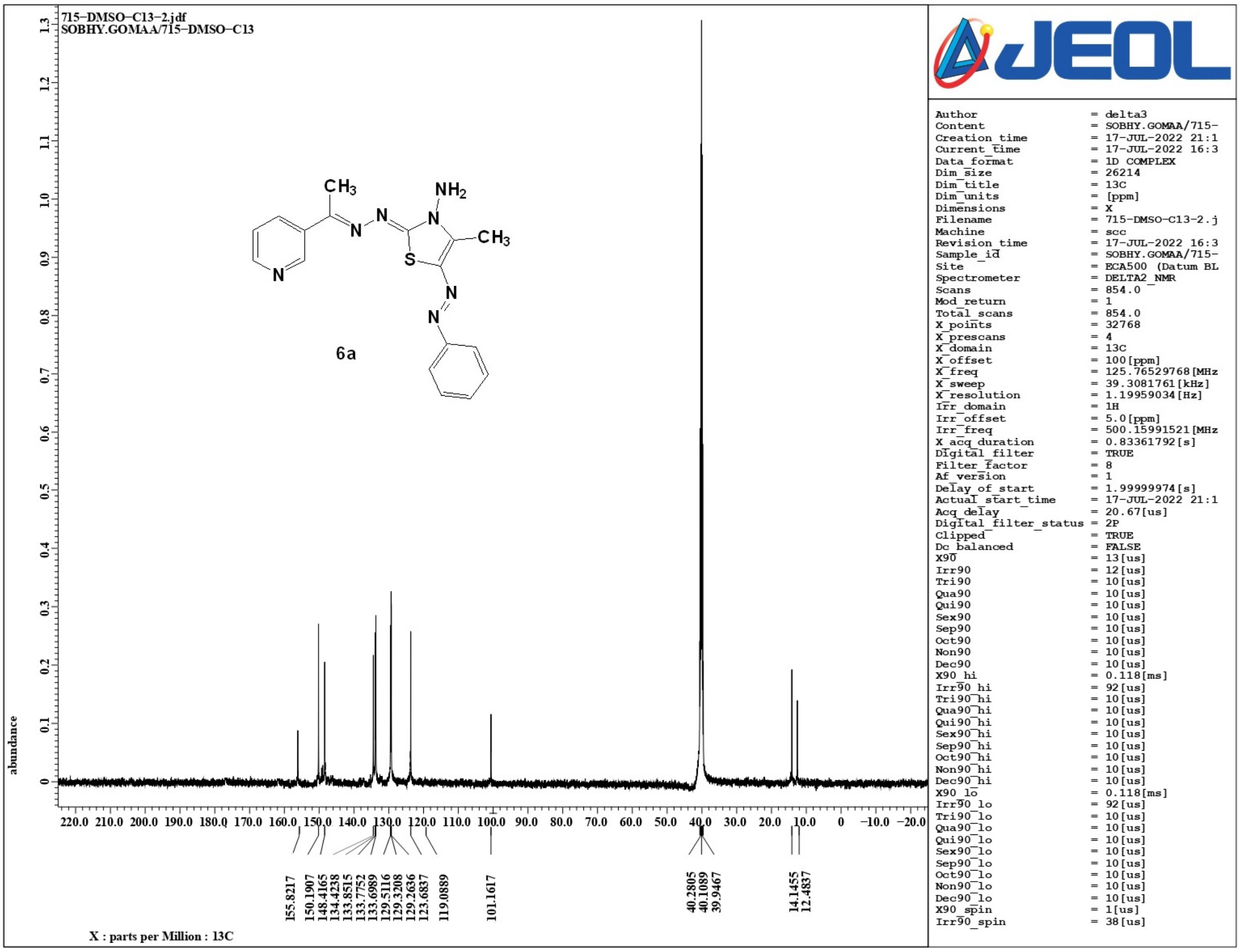

^{13}C-NMR spectra of compound **6a**

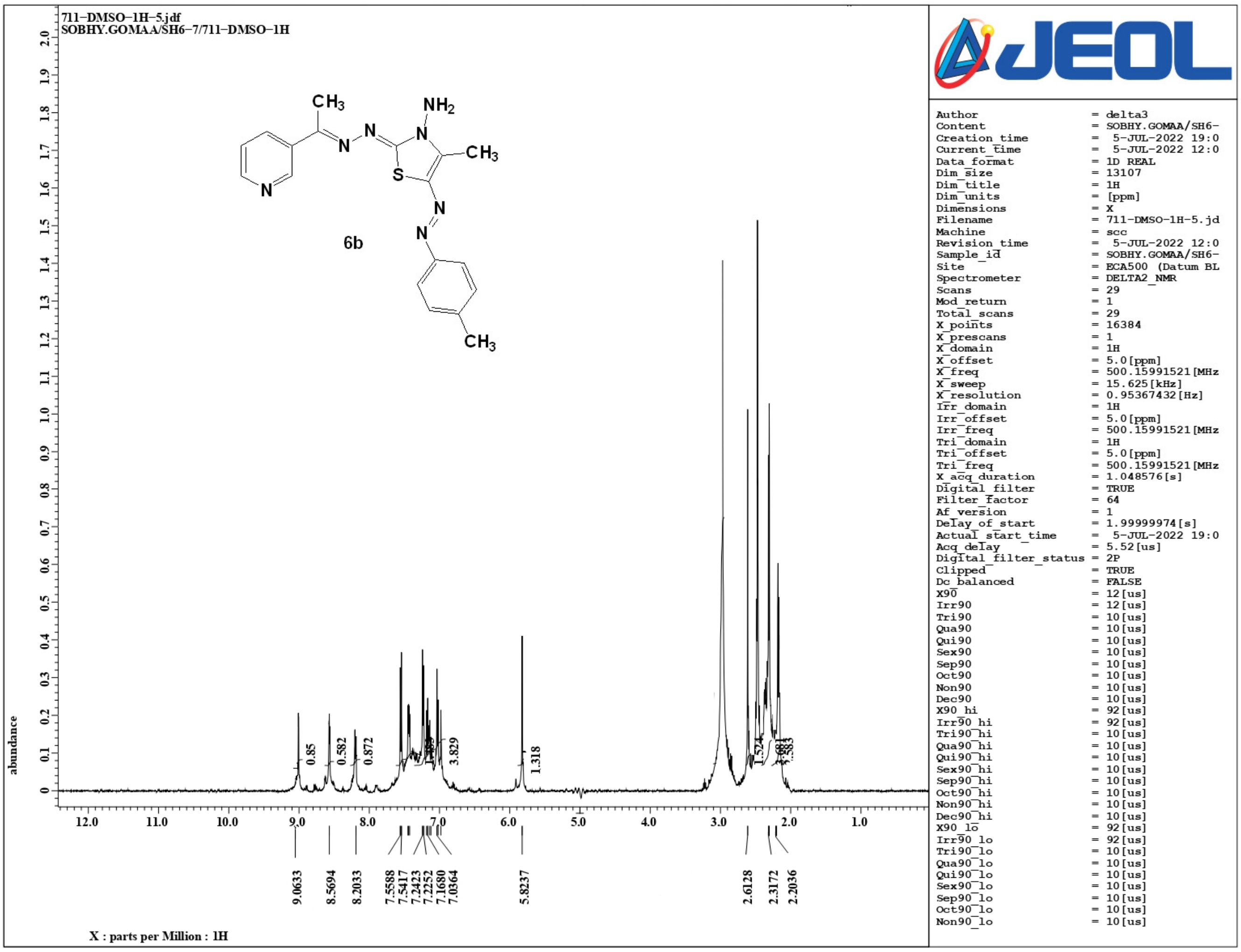

^{1}H-NMR spectra of compound **6b**

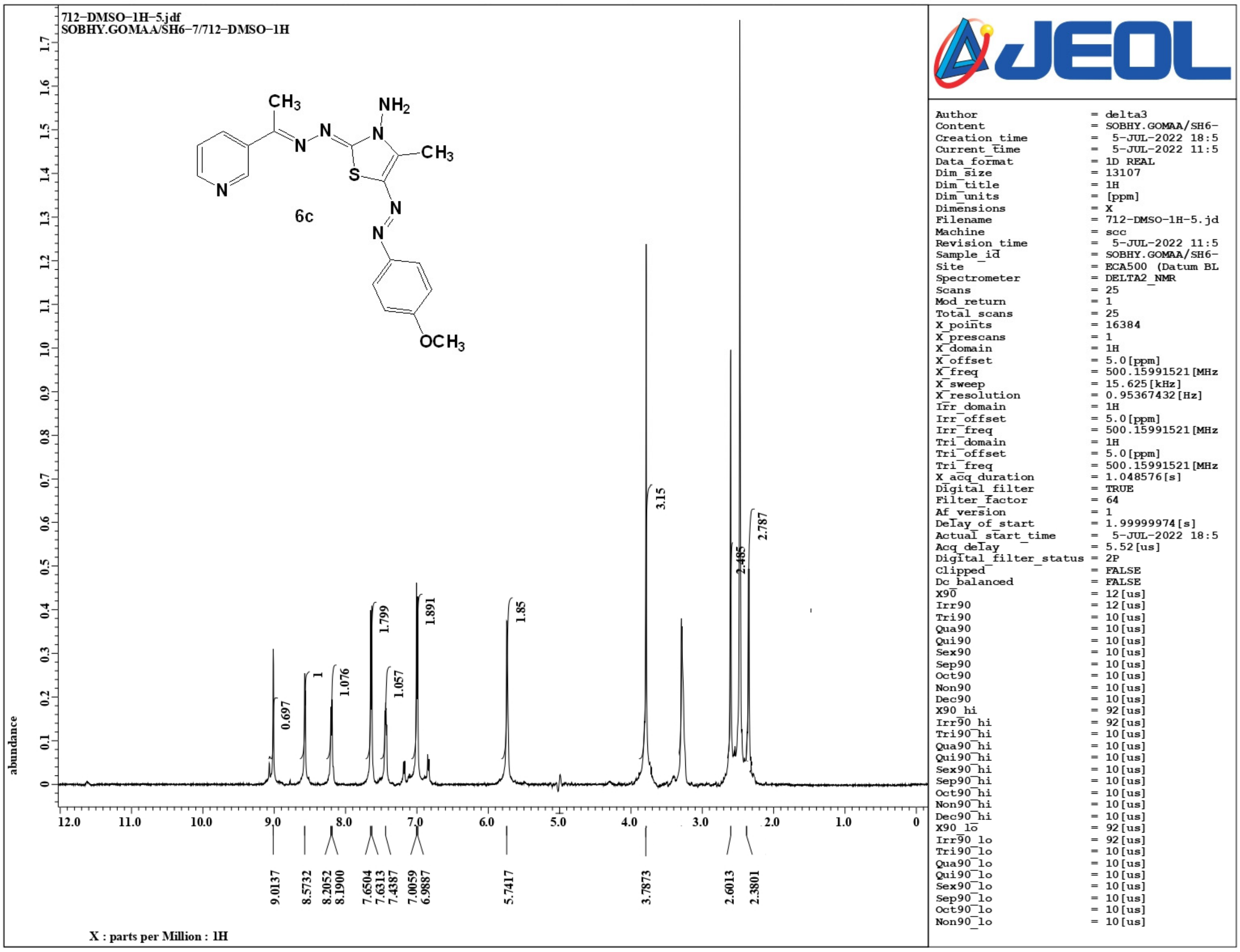

^{1}H-NMR spectra of compound **6c**

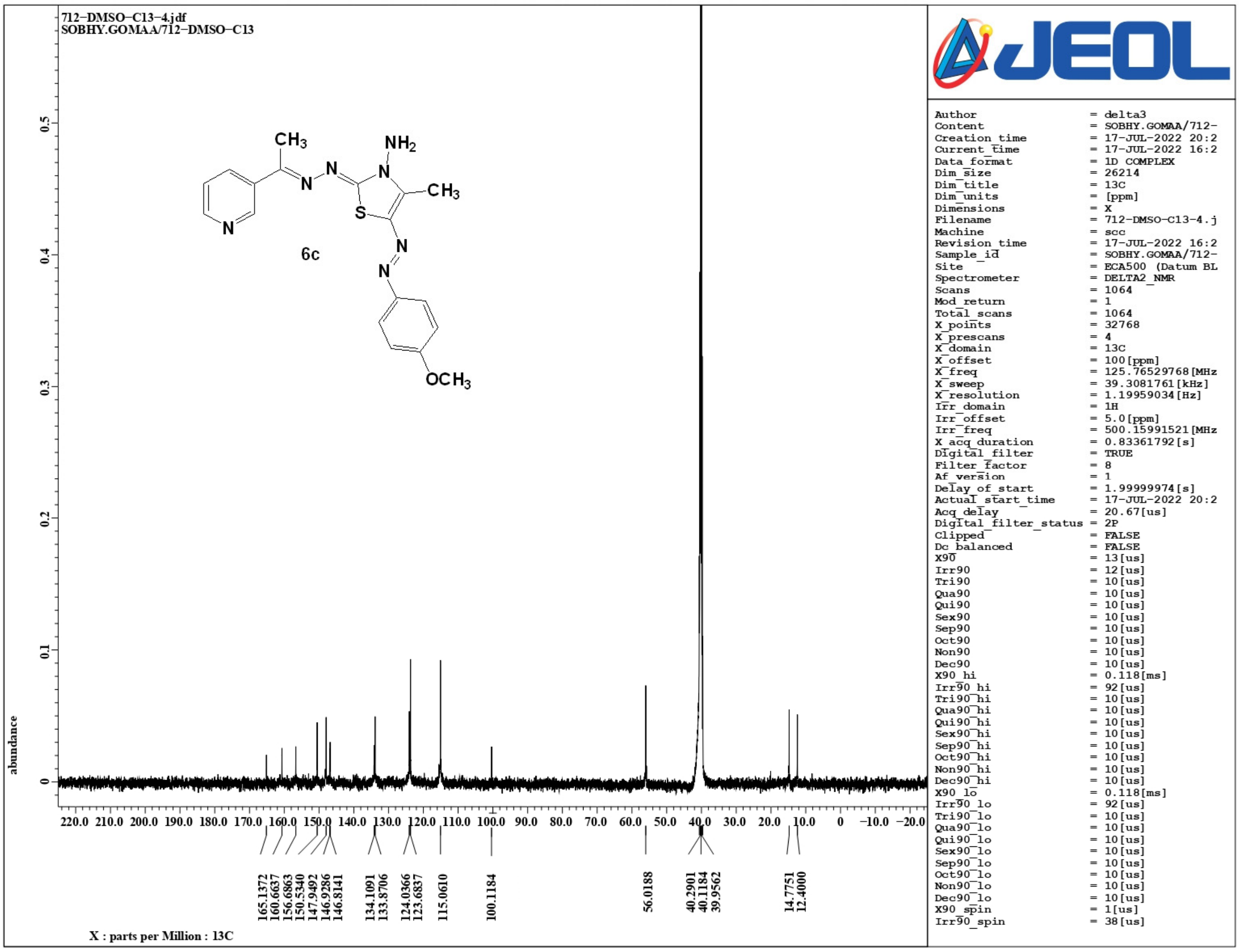

^{13}C-NMR spectra of compound **6c**

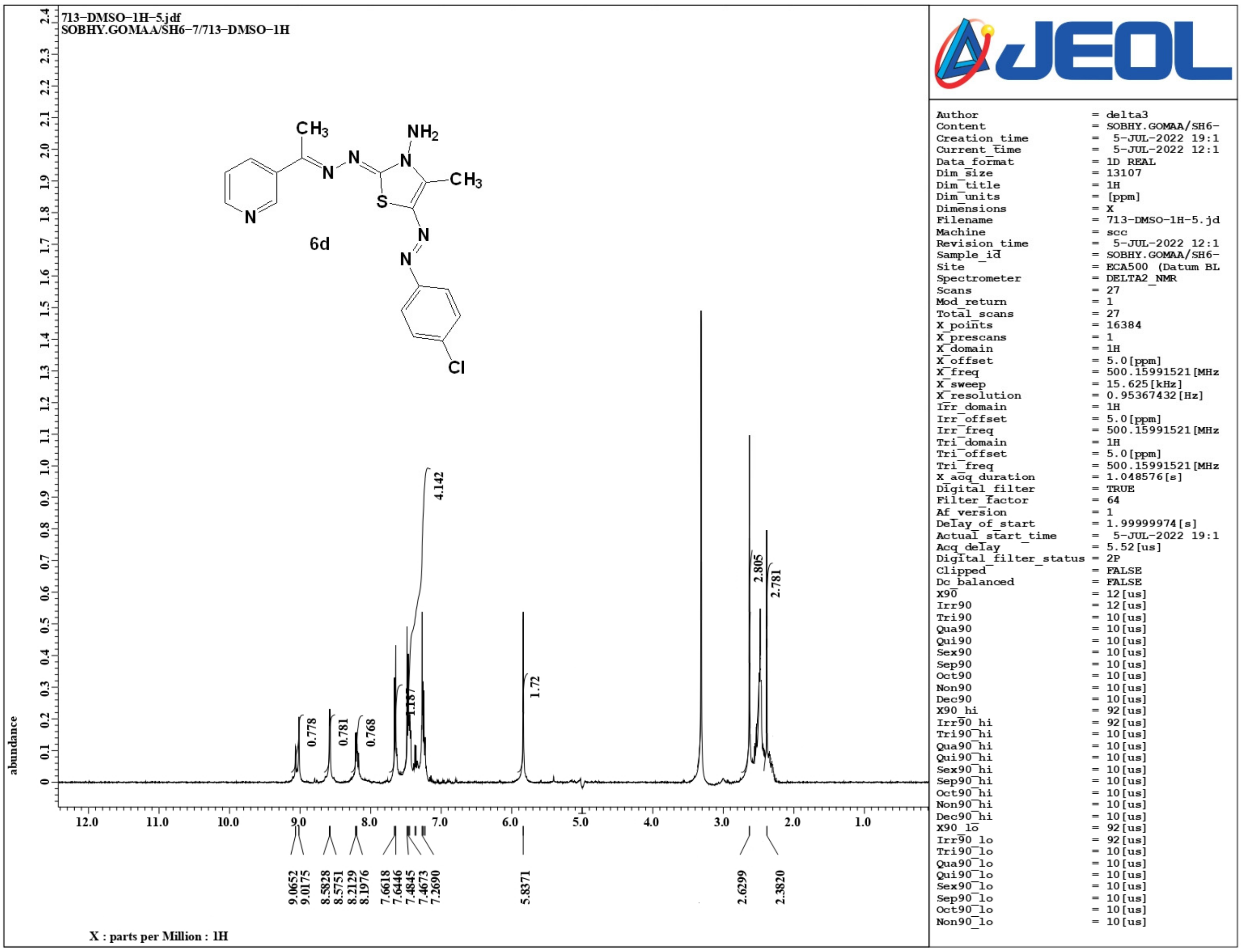

^{1}H-NMR spectra of compound **6d**

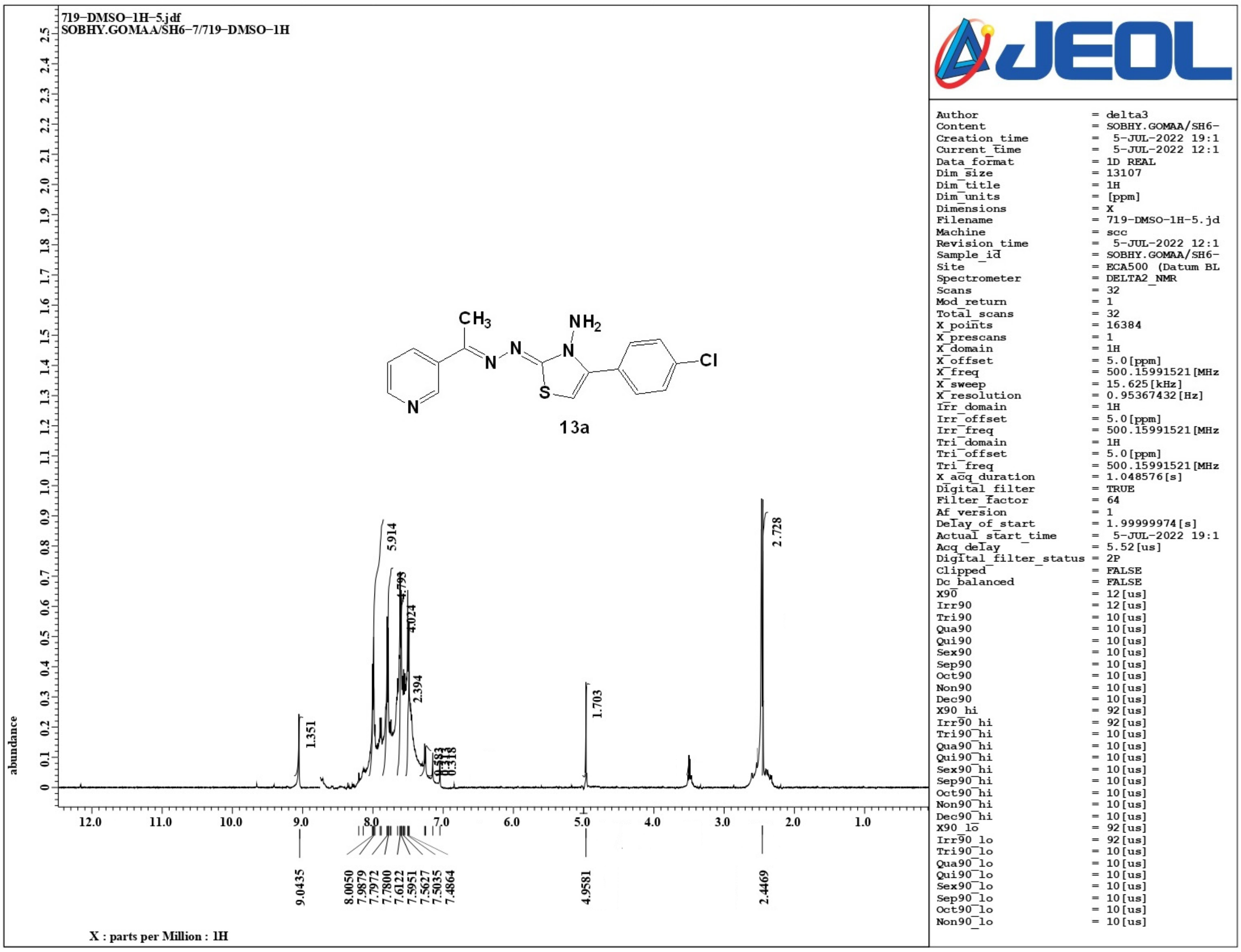

^{1}H-NMR spectra of compound **13a**

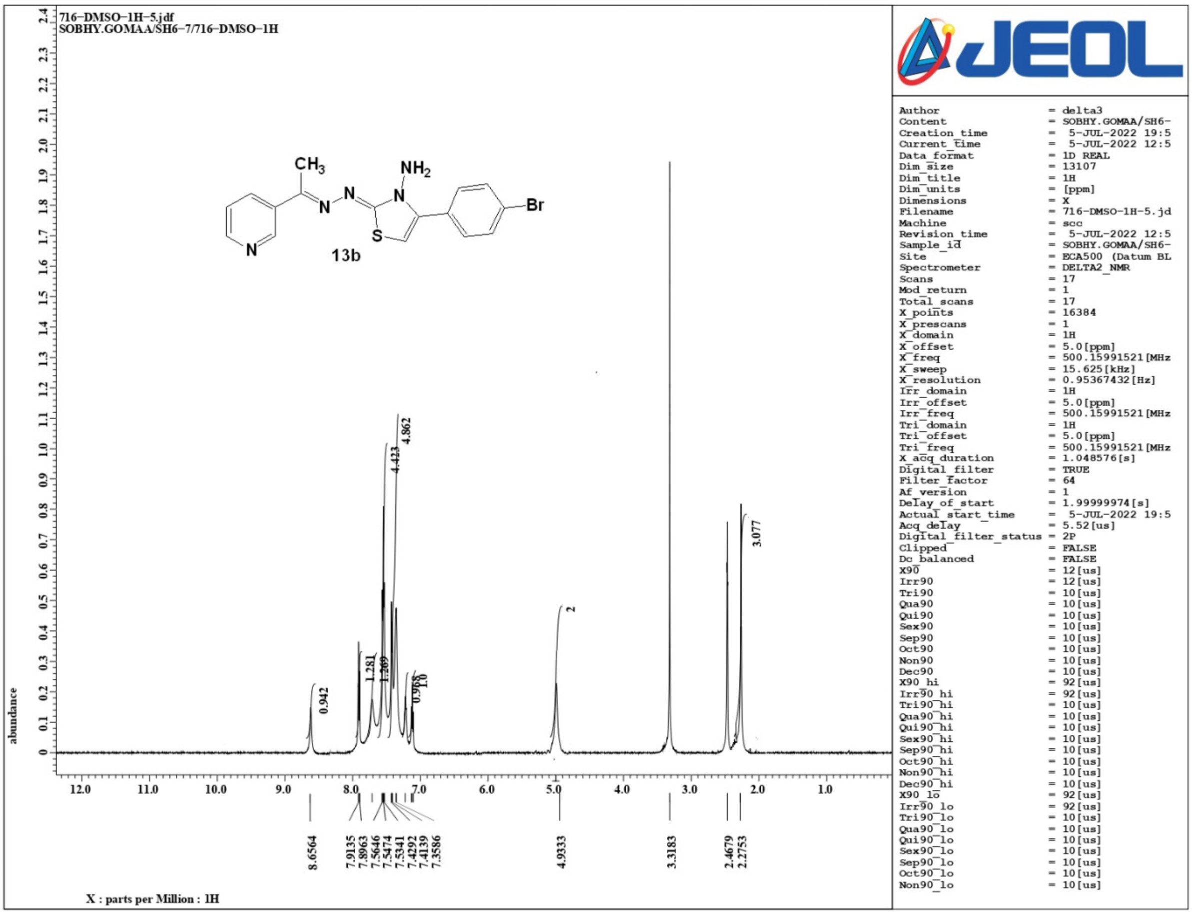

^{1}H-NMR spectra of compound **13b**

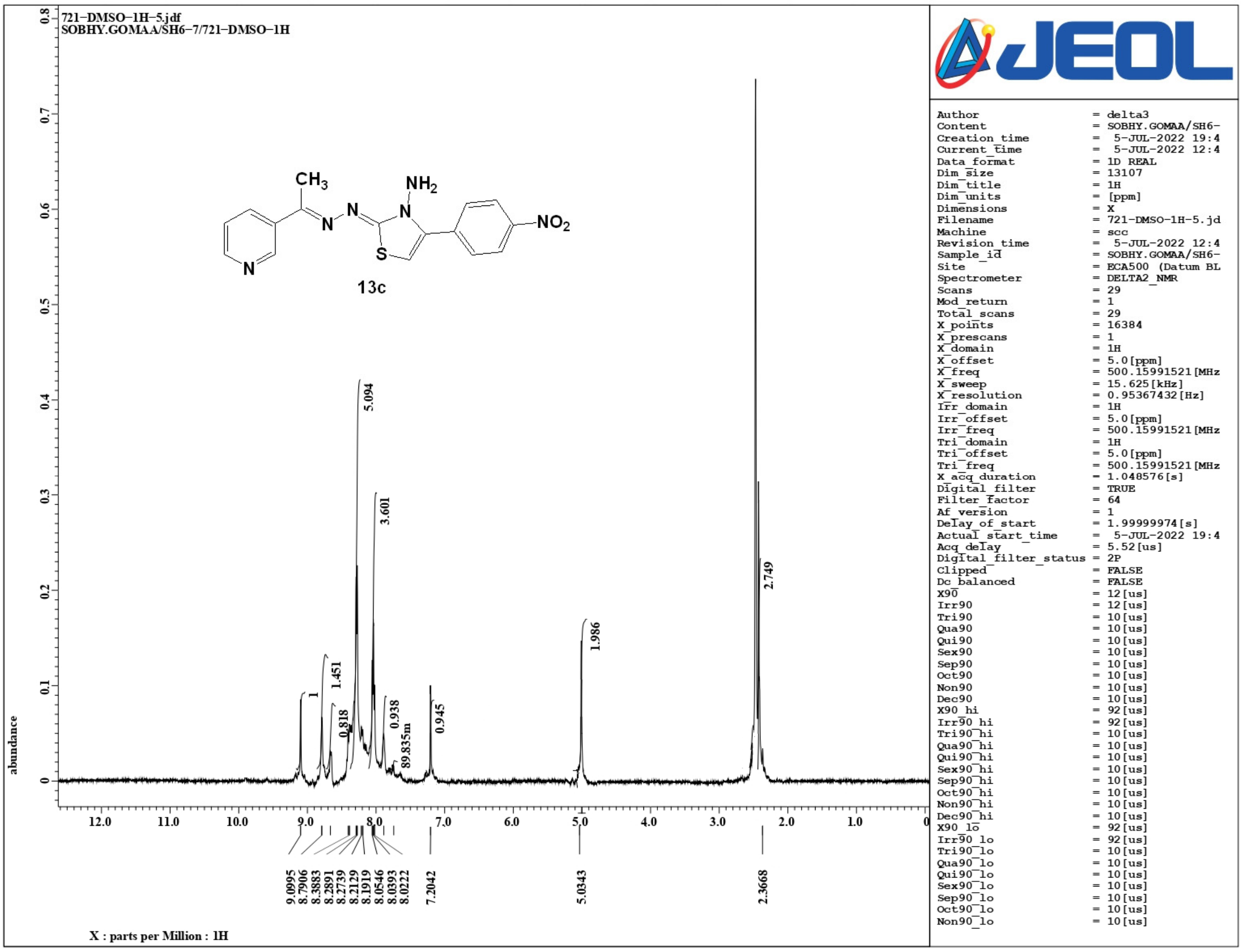

^{1}H-NMR spectra of compound **13c**

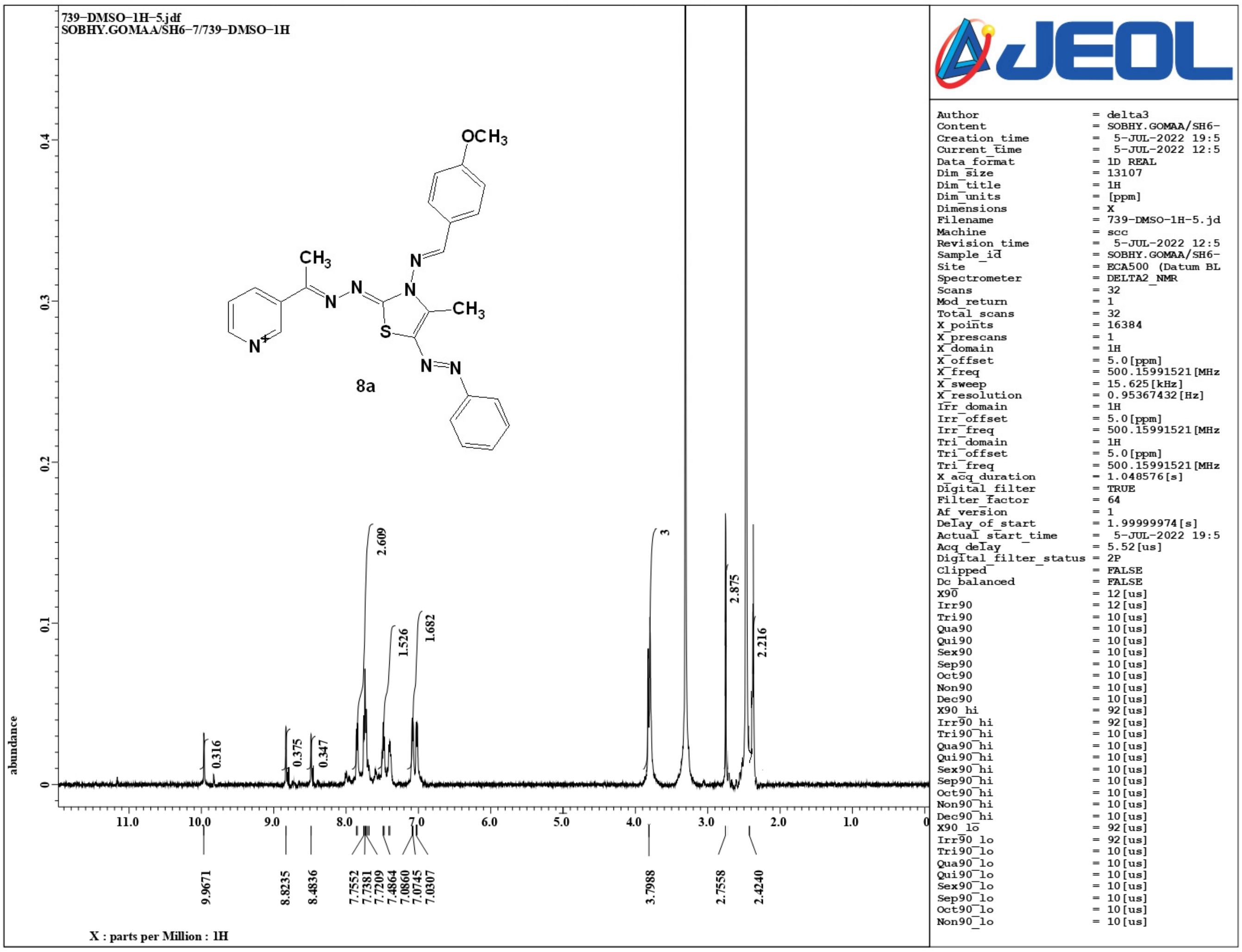

^{1}H-NMR spectra of compound **8a**

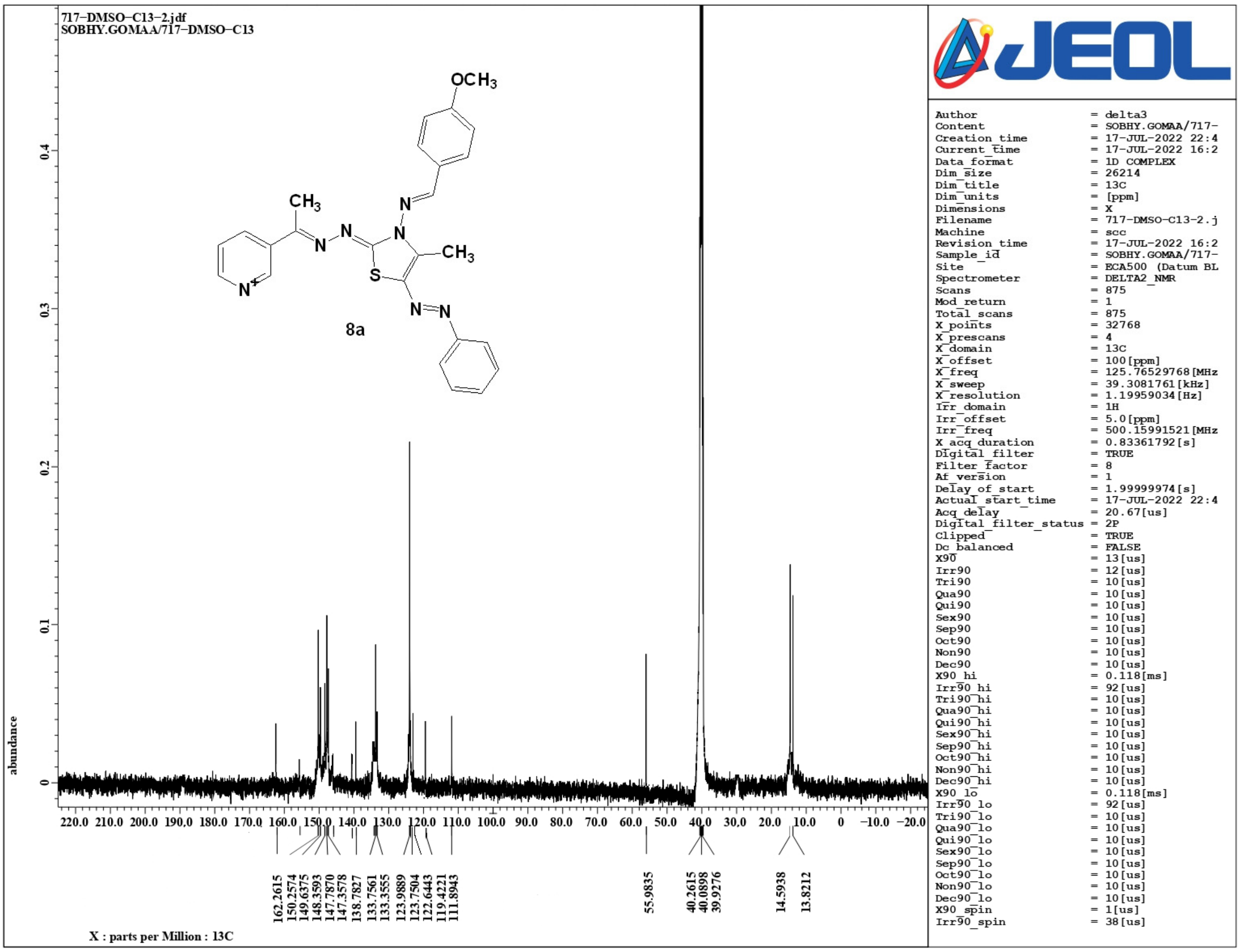

^{13}C-NMR spectra of compound **8a**

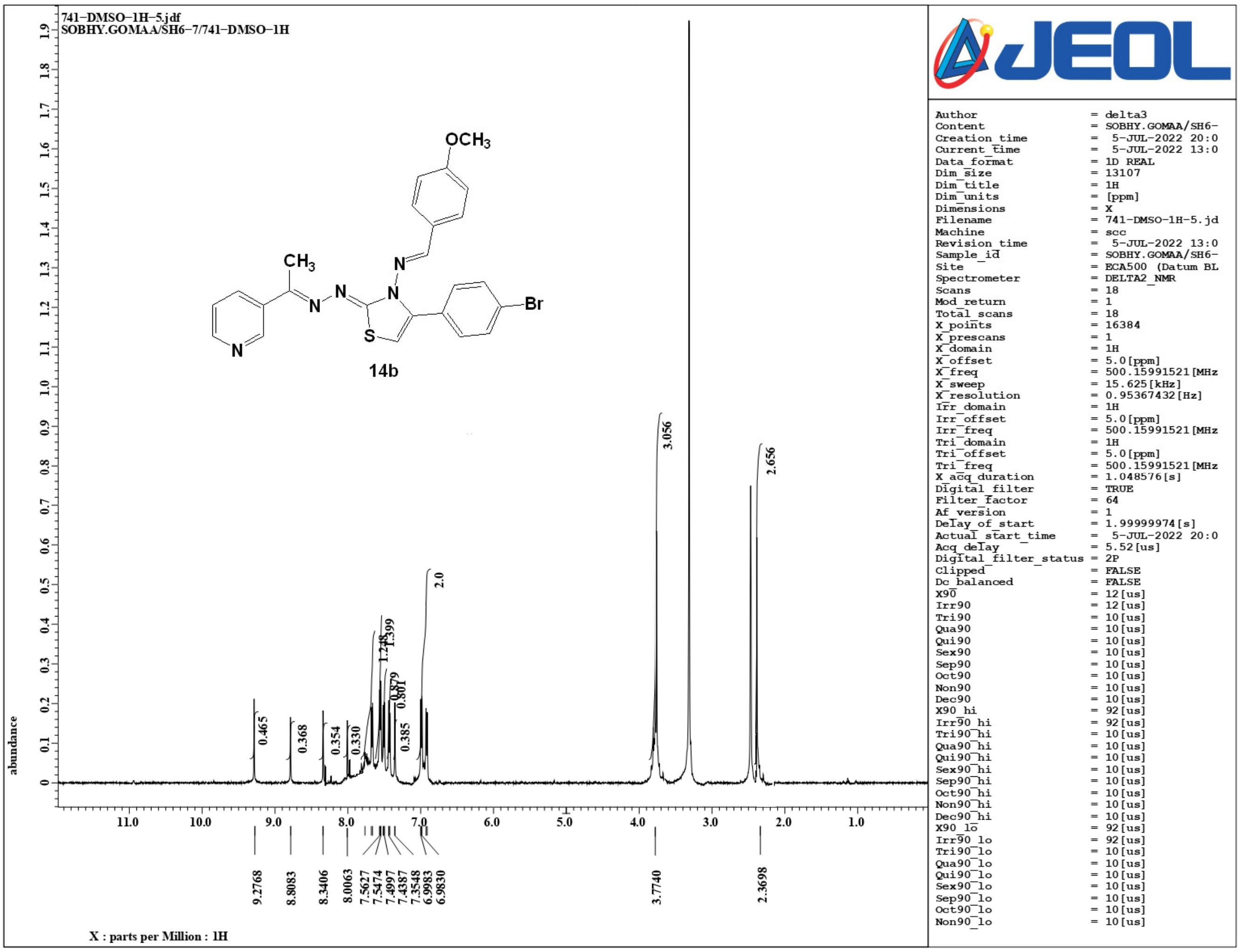

^{1}H-NMR spectra of compound **14b**

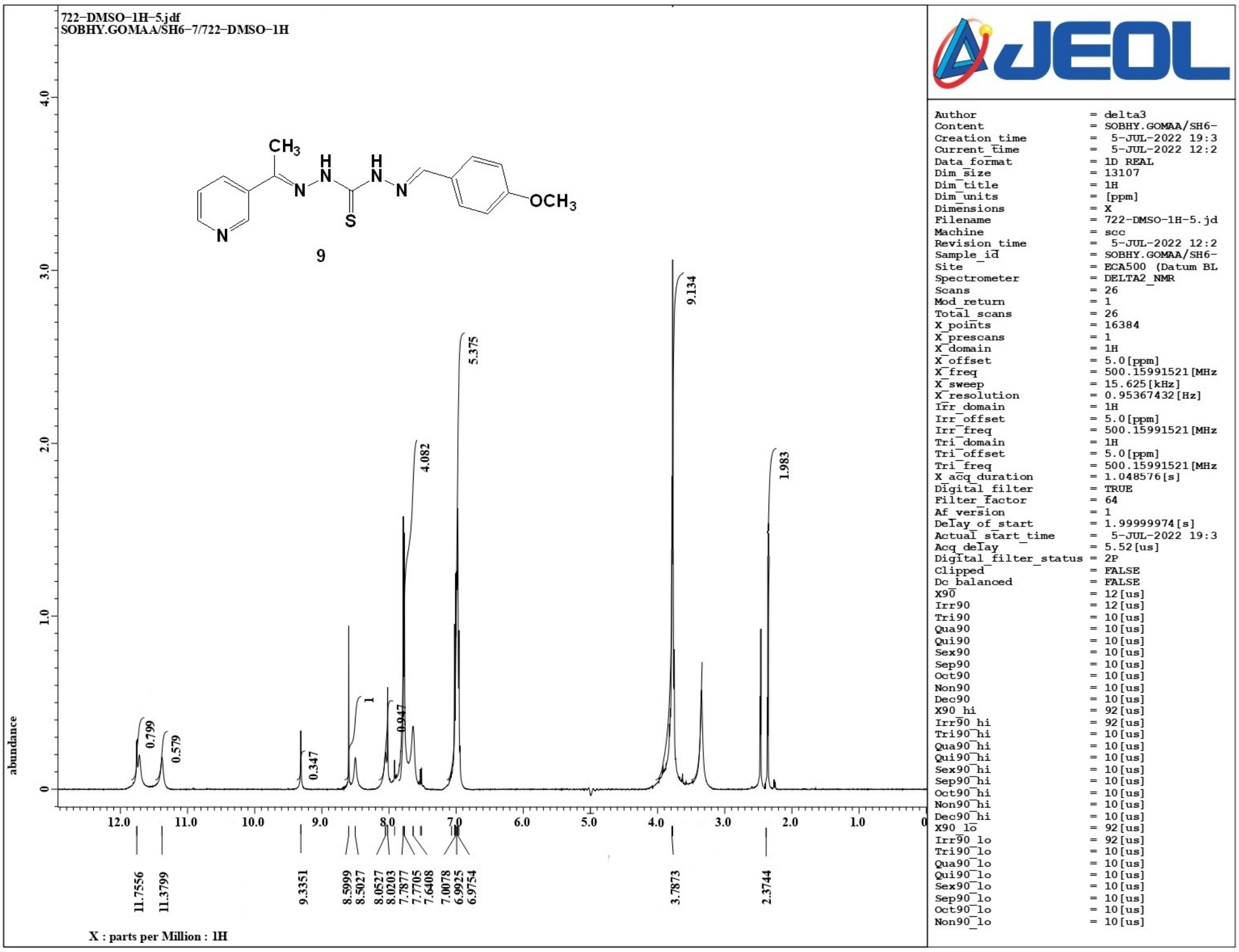

^{1}H-NMR spectra of compound **9**
